# Dietary fiber consumption and outcomes of different cancers: an umbrella review

**DOI:** 10.29219/fnr.v69.11034

**Published:** 2025-01-03

**Authors:** Xingyu He, Jiayi Hou, Lei Liu, Xin Chen, Lijie Zhang, Caojia Pang, Yu Tong, Hongling Li, Feng Chen, Rong Peng, Zheng Shi

**Affiliations:** 1 Clinical Medical College & Affiliated Hospital of Chengdu University, Chengdu University, Sichuan, China; 2 Basic Medical School, Chengdu University, Chengdu, Sichuan, China; 3 Zunyi Medical University, School of Pharmacy, Zunyi, Guizhou, China

**Keywords:** dietary fiber, cancer prevention, systematic reviews as topic, risk reduction behavior, observational studies

## Abstract

**Background:**

The relationship between dietary fiber intake and cancer outcomes, including incidence, recurrence, and mortality, is crucial for understanding cancer prevention strategies.

**Methods:**

An umbrella review was conducted, analyzing existing systematic reviews and meta-analyses from PubMed, Embase, and the Cochrane Database of Systematic Reviews. This included data from 26 meta-analyses based on 2,107 unique articles, covering 52 observational study outcomes. The quality of the studies was assessed using the AMSTAR 2 tool.

**Results:**

High fiber intake significantly lowers the risk of cancers affecting the digestive, reproductive, and urinary systems, including esophageal adenoma, squamous cell carcinoma, gastric, pancreatic, colon, rectal, colorectal adenoma, breast, ovarian, endometrial, prostate, renal cell, and bladder cancers. Findings estimated that the risk of Colon cancer between total dietary fiber (TDF) was 0.74 (95% confidence interval [CI]: 0.67–0.82), and the risk of Colorectal cancer between TDF was 0.88 (95% CI: 0.82–0.94). TDF was also found to be protective against Barrett’s esophagus and esophagus cancer, esophageal adenomas, and esophagus squamous cell carcinoma, with effect sizes of 0.52 (95% CI: 0.43–0.64), 0.50 (95% CI: 0.37–0.67), and 0.53 (95% CI: 0.31–0.90), respectively. Conversely, increased intake of cereal fiber was associated with a higher incidence of renal cell carcinoma and endometrial cancer. Dose–response analyses revealed that increments of 2.5, 5, or 10 g per day in dietary fiber could lead to different levels of risk reduction for these cancers. Meta-regression suggested an optimal fiber intake range of 7–36 g per day for colon cancer prevention. However, the overall study quality was predominantly rated as ‘very low’.

**Conclusions:**

Higher dietary fiber intake is linked to reduced cancer risk and improved outcomes. These findings highlight dietary fiber’s importance in cancer prevention and care.

## Popular scientific summary

- **What’s new:** This umbrella review demonstrates a strong inverse relationship between high dietary fiber intake and the risk of several cancers, particularly in the digestive, reproductive, and urinary systems.
- **Implications:** Increasing daily fiber consumption, within an optimal range of 7–36 g, could significantly reduce colon cancer incidence, recurrence, and mortality. Public health strategies should emphasize dietary fiber intake as a key component of cancer prevention and care.

Dietary fiber, a fundamental component of human health and often referred to as the ‘seventh nutrient’, is derived from various sources such as grains, vegetables, fruits, nuts, and others (1). It is classified into soluble dietary fiber (SDF) and insoluble dietary fiber (IDF) types based on water solubility and origin. IDF primarily consists of cell wall components like cellulose, lignin, and hemicellulose, while SDF encompasses non-cellulosic polysaccharides like gums and mucilages (2). Research indicates that higher dietary fiber intake may reduce the risk of cancers such as colorectal, breast, and endometrial cancer (3–5). The Food and Drug Administration (FDA) recognizes the health benefits, stating that higher fiber consumption can potentially decrease cancer incidence (6). Recently, systematic reviews and meta-analyses have been published, revealing mixed findings. Some studies suggest dietary fiber may reduce mortality in cancer patients, while others report no effect or even potential harm (7–10). An umbrella review, which synthesizes all available evidence, can help address these discrepancies. This review aims to provide recommendations for the use of dietary fiber in the context of cancer care.

## Methods

### Umbrella review methods

A detailed search was performed to locate systematic reviews and meta-analyses that explore the connection between dietary fiber consumption and cancer risk. Data from these selected studies were meticulously extracted to analyze the associations. The included studies were thoroughly evaluated with the AMSTAR 2 tool to verify the reliability and validity of the results. This research was registered with PROSPERO, under the registration number CRD42023445992, to ensure transparency and adherence to established protocols.

### Literature search

An in-depth literature search was performed until August 9, 2024, using databases such as PubMed, Embase, and the Cochrane Database of Systematic Reviews. The detailed search strategy employed for this review is documented in Supplementary Material Table S1.

### Eligibility criteria

Only English-language reviews focusing on adults aged 18 and above were included. The original studies within the systematic reviews and meta-analyses consisted of randomized controlled trials, cohort studies, and case–control studies. Meta-analyses were included if they provided relative risk, odds ratio (OR), or risk ratio (RR) with 95% confidence intervals (CIs), assessing the impact of fiber interventions on cancer risk or mortality. Individual data extraction was conducted for each distinct cancer outcome. When multiple meta-analyses from the same population with consistent methodologies and findings were present, the one with the largest sample size was given priority, unless the study’s quality was questionable. Dose-response analyses were also taken into account, with preference given to the most recent study; if unavailable, both earlier and later studies were considered. The study was inclusive, without any gender or racial restrictions.

This umbrella review applied stringent inclusion and exclusion criteria to ensure high methodological standards. We excluded non-systematic reviews, narrative reviews, studies based on animal cells, and umbrella reviews. Only systematic reviews and meta-analyses evaluating the effect of dietary fiber on cancer outcomes in human populations were included. Studies focusing on complex interventions that combined dietary fiber with other dietary patterns or food products containing non-fiber ingredients were also excluded. Furthermore, we removed duplicate meta-analyses that reported identical findings if they were based on the same original data but had a smaller sample size or lower quality. To ensure relevance, books, editorials, letters, and studies with incomplete abstracts or inadequate data for evaluating cancer outcomes were excluded. A detailed list of exclusions is available in Supplementary Material Table S2.

### Data extraction

Initially, two reviewers (XH and XC) independently assessed titles and abstracts based on set inclusion and exclusion criteria, followed by a full-text review of the qualifying articles. Any discrepancies between the reviewers were resolved through discussion or by consulting a third researcher (RP). The extracted information included the first author’s name, publication year, country, and types of dietary fiber (total, soluble, insoluble, vegetable, fruit, cereal, legume, and bran) as well as intake levels, duration, and cancer type. The outcomes assessed were cancer incidence, recurrence, all-cause mortality, and specific mortality. We documented key study design features, including type, sample size, and effect measures such as RR, OR, and hazard ratio (HR), all with 95% CIs. Heterogeneity was analyzed using the I^2^ statistic and Cochran’s Q test *P*-value. The chosen effect model was documented, and potential publication bias was assessed. For dose-response assessments, relevant dietary fiber intake levels were extracted.

### Methodology quality

Two reviewers (XH and JH) evaluated the methodological integrity of the included studies through the utilization of AMSTAR, a validated and reliable measurement tool for evaluating the quality of systematic reviews and meta-analyses. The methodological integrity of the included studies was evaluated using the AMSTAR 2 tool, which consists of 16 domains covering key quality measures, including the risk of bias, study selection, and data synthesis. We focused particularly on seven critical domains (questions 2, 4, 7, 9, 11, 13, and 15), which directly assess the robustness of systematic reviews. Studies were graded as ‘high’ quality if they had no critical flaws, ‘moderate’ if they had multiple non-critical flaws, ‘low’ for one critical flaw, and ‘very low’ if they exhibited multiple critical flaws (11).

### Data analysis

The association between dietary fiber and cancer was analyzed by extracting effect sizes and 95% CIs from the chosen studies. We endeavored to elucidate the sources of statistical heterogeneity, employing meta-regression to identify effect modification. This study compiles data from a systematic review of pertinent original research articles focusing on significant outcomes. A total of 21 studies, meeting established criteria, were included, providing data on daily fiber intake (g/day) for both experimental and control groups, RR (HR, and OR), and 95% CIs. For each study, we computed the log OR alongside its standard error. To evaluate the influence of dietary fiber intake on the occurrence of significant findings, we employed a linear regression model, differentiating between experimental and control groups. Our analysis employed a random effects model (Random Effects Model, RE), and we reported the Z-distribution and log OR for a comprehensive understanding.

## Results

### Characteristics of meta-analyses

**Fig. 1:**
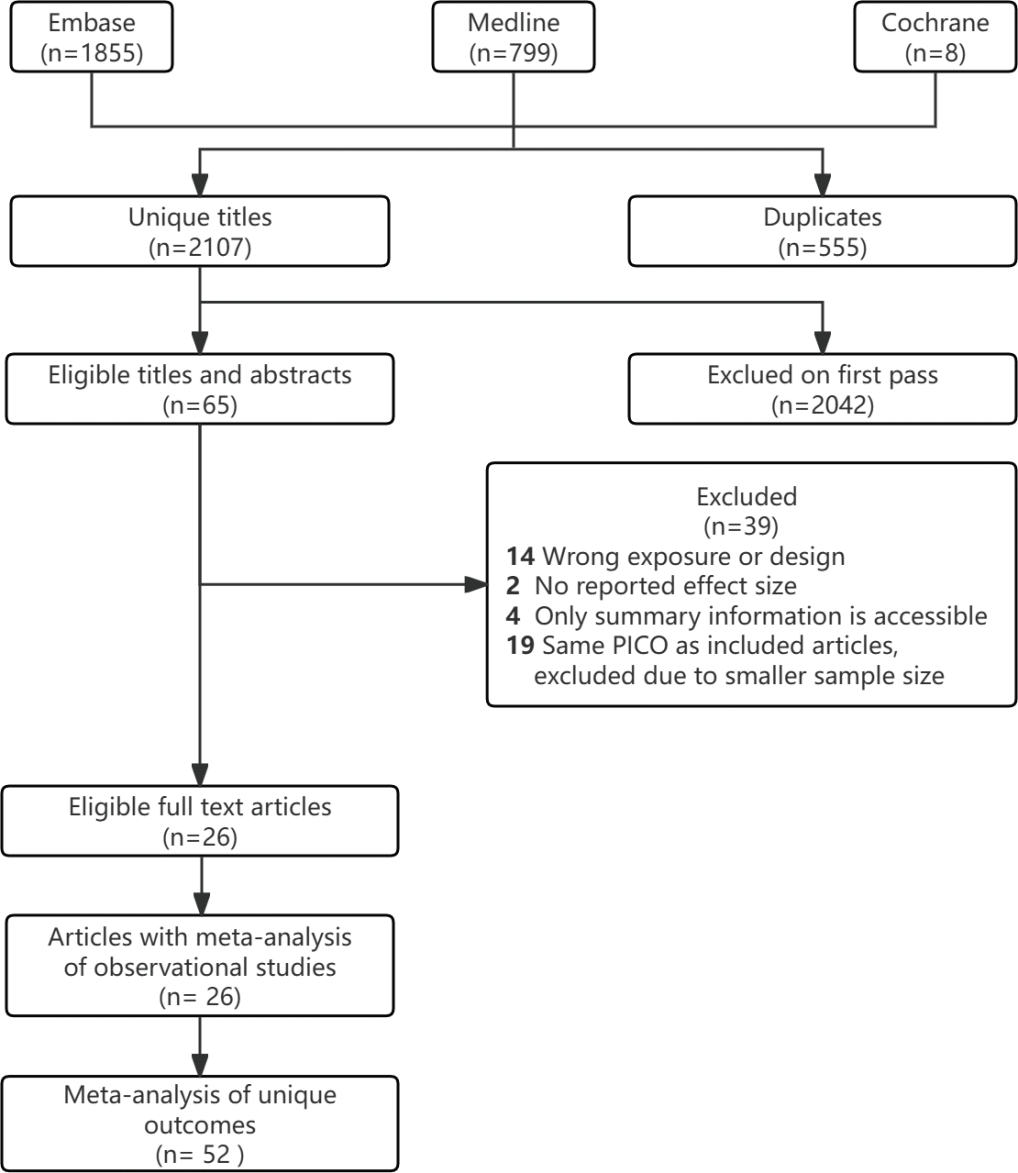
Literature screening process.

**Fig. 2:**
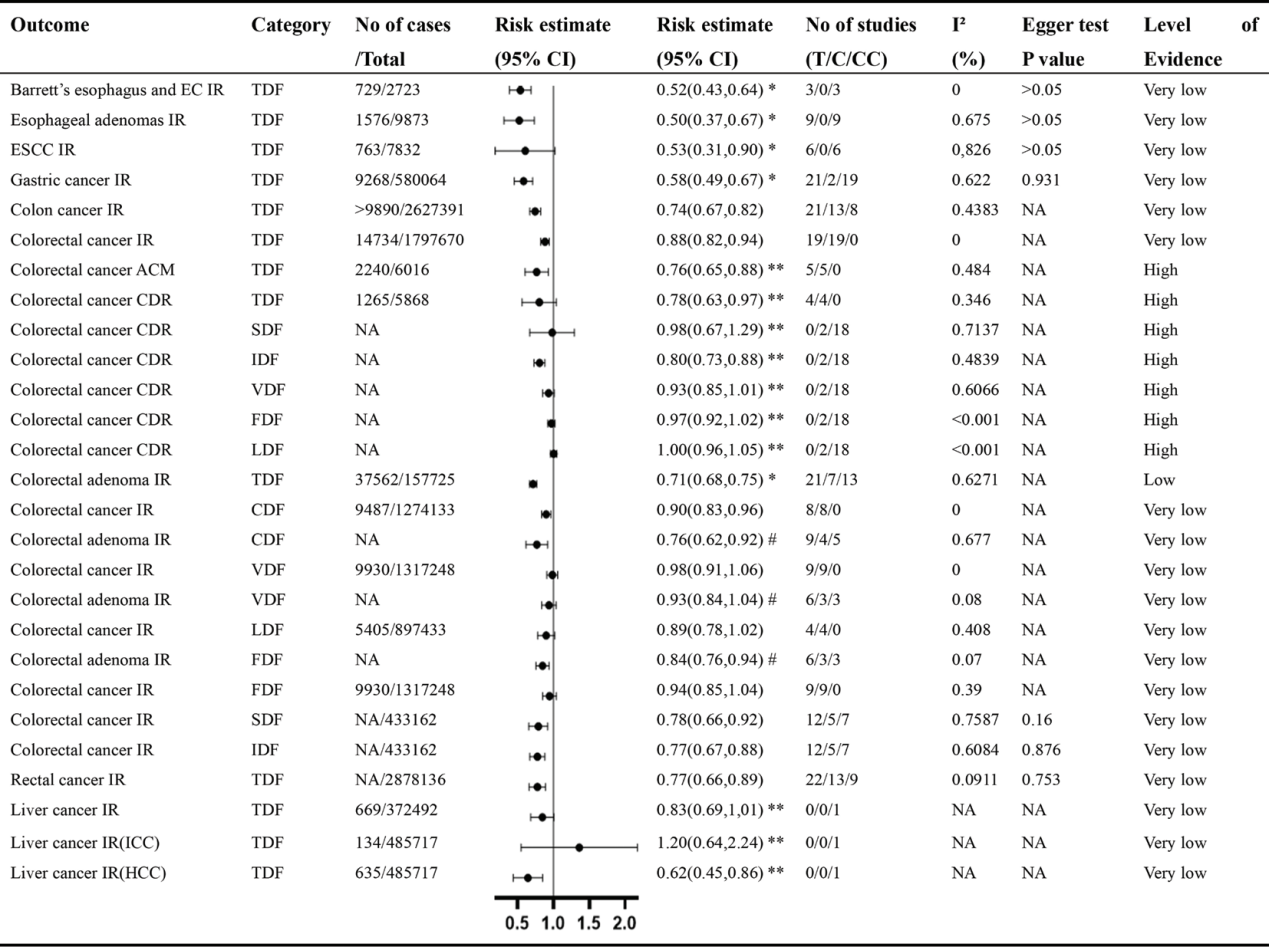
Non-dose-response relationship between various types of dietary fiber intake and various gastrointestinal cancers. Comparisons are highest versus lowest, estimates are relative risks, and effect models are random unless noted otherwise. T, total study; C, cohort study; CC, case–control study; CI, confidence interval; NA, not available; EC, esophagus cancer; ESCC, esophagus squamous cell carcinoma; ICC, intrahepatic cholangiocarcinoma; HCC, hepatocellular carcinoma; IR, incident rate; SM, specific mortality rate; ACM, all-cause mortality rate; TDF, total dietary fiber; CDF, cereal dietary fiber; VDF, vegetable dietary fiber; LDF, legume dietary fiber; FDF, fruit dietary fiber; SDF, soluble dietary fiber; IDF, insoluble dietary fiber; *Odds ratio; **Hazard ratio; #summary relative risks.

**Fig. 3:**
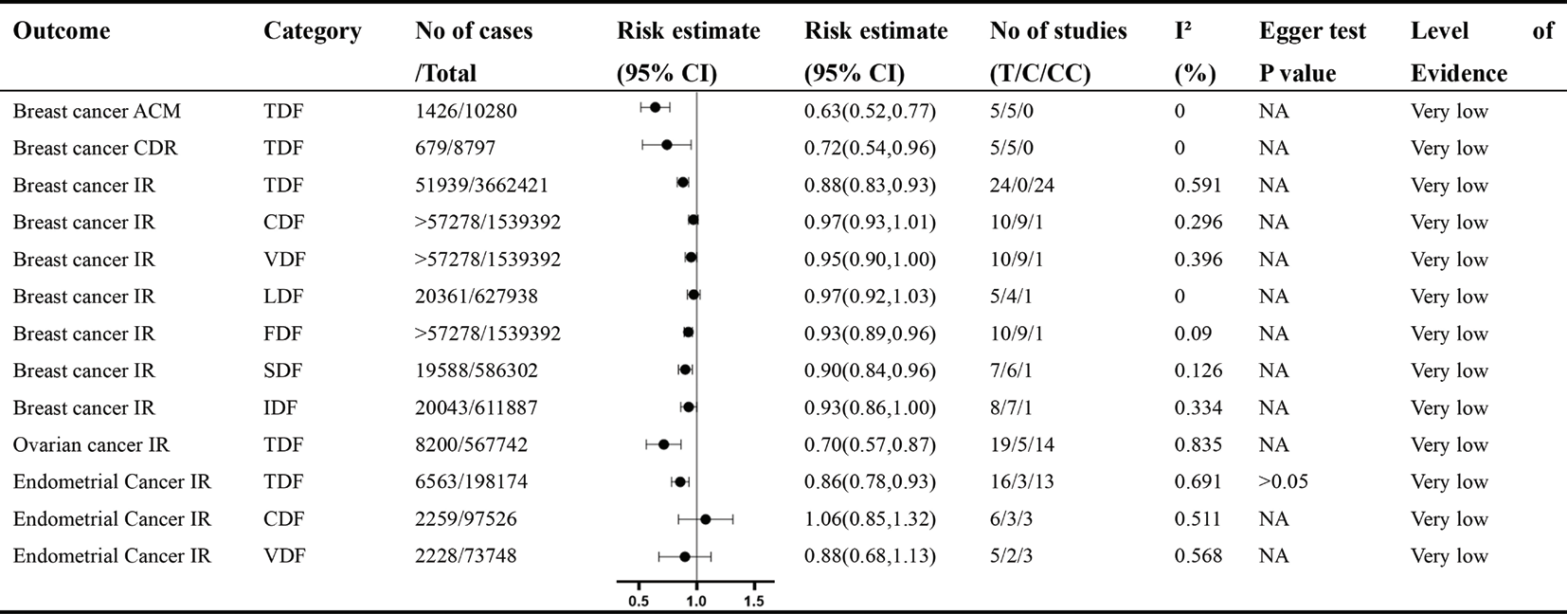
Non-dose-response relationship between various types of dietary fiber intake and various reproductive cancers. Comparisons are highest versus lowest, estimates are relative risks, and effect models are random unless noted otherwise. T, total study; C, cohort study; CC, case–control study; CI, confidence interval; NA, not available; IR, incident rate; CDR, crude death rate; ACM, all-cause mortality rate; TDF, total dietary fiber; CDF, cereal dietary fiber; VDF, vegetable dietary fiber; LDF, legume dietary fiber; FDF, fruit dietary fiber; SDF, soluble dietary fiber; IDF, intolerable dietary fiber.

**Fig. 4:**
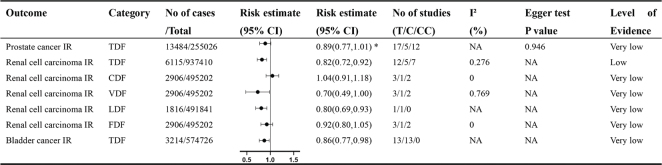
Non-dose-response relationship between various types of dietary fiber intake and various genitourinary cancers. Comparisons are highest versus lowest, estimates are relative risks, and effect models are random unless noted otherwise. T, total study; C, cohort study; CC, case–control study; CI, confidence interval; NA, not available; IR, incident rate; TDF, total dietary fiber; CDF, cereal dietary fiber; VDF, vegetable dietary fiber; LDF, legume dietary fiber; FDF, fruit dietary fiber; *Odds ratio.

**Fig. 5:**
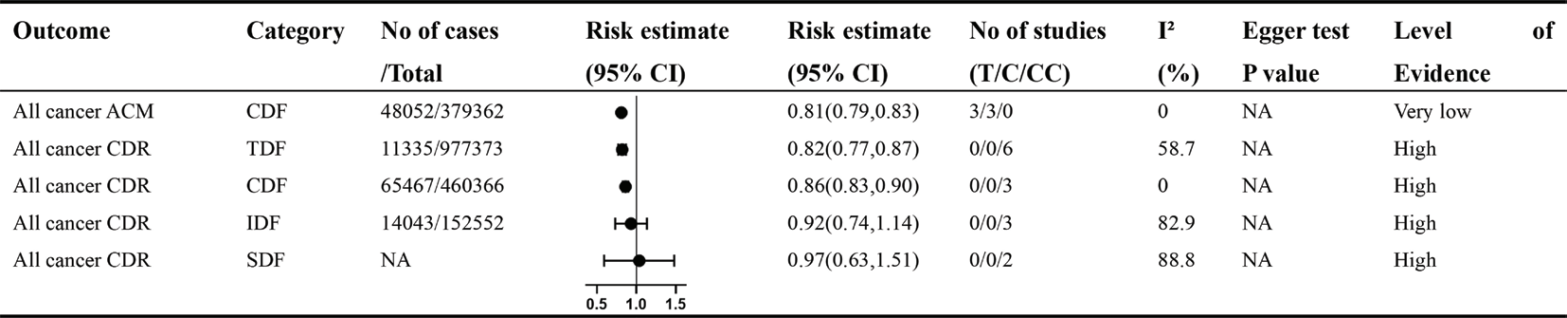
Non-dose-response relationship between various types of dietary fiber intake and all cancers. Comparisons are highest versus lowest, estimates are relative risks, and effect models are random unless noted otherwise. T, total study; C, cohort study; CC, case–control study; CI, confidence interval; NA, not available; CDR, crude death rate; ACM, all-cause mortality rate; TDF, total dietary fiber; CDF, cereal dietary fiber.

**Fig. 6:**
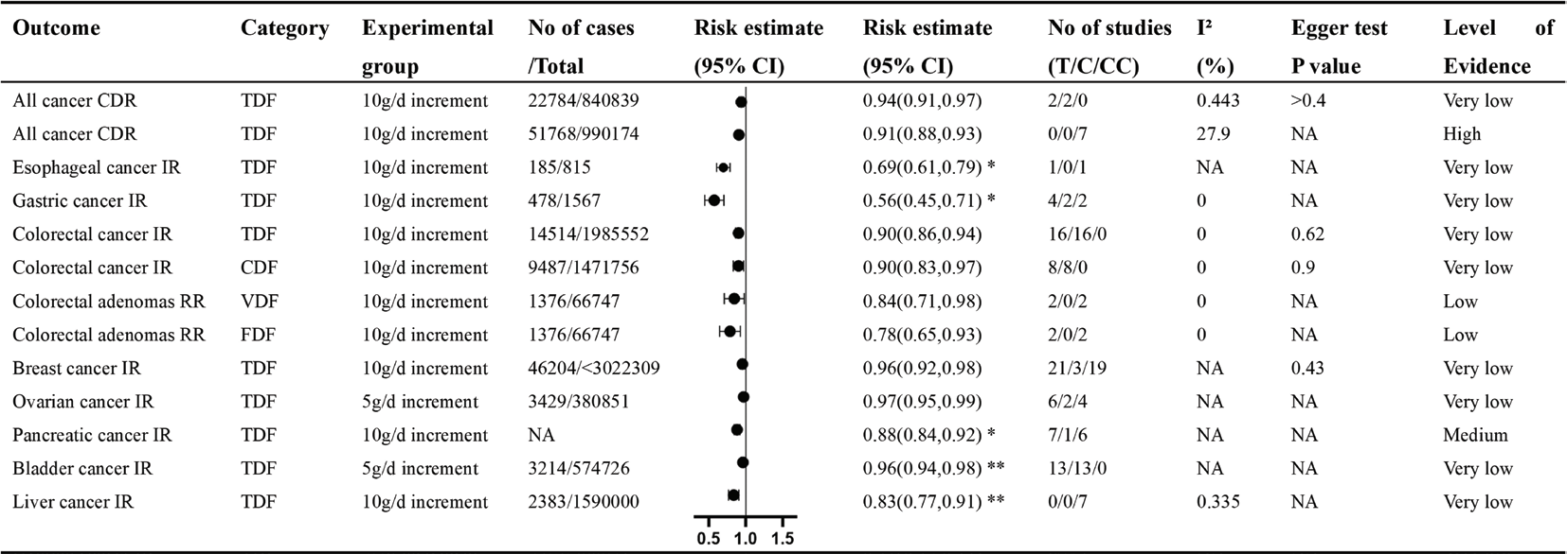
Dose-response relationship between dietary fiber intake and multiple cancers. T, total study; C, cohort study; CC, case–control study; CI, confidence interval; NA, not available; CDR, crude death rate; IR, incident rate; RR, relapse rate; TDF, total dietary fiber; CDF, cereal dietary fiber; VDF, vegetable dietary fiber; FDF, fruit dietary fiber; *Odds ratio; ** Hazard ratio.

The initial search yielded 2,107 unique articles, which were narrowed down to 65 after the initial screening. Further exclusions were made due to incorrect exposure or design (*n* = 14), unreported effect sizes (*n* = 2), lack of detailed data (*n* = 4), and duplication of outcomes and studies (*n* = 19). This process resulted in the final selection of 26 meta-analyses, all of which were observational studies. A total of 52 distinct outcomes were extracted from these studies (Fig. 1). The primary focus was on colorectal cancer (*n* = 19), followed by breast cancer (*n* = 9), renal cell carcinoma (RCC) (*n* = 5), esophageal cancer (ESCA) (*n* = 3), all cancers combined (*n* = 5), endometrial cancer (*n* = 3), liver cancer (*n* = 3), gastric cancer (*n* = 1), ovarian cancer (*n* = 1), adenocarcinomas (*n* = 1), bladder cancer (*n* = 1), and rectal cancer (*n* = 1). The studies analyzed various types of dietary fiber, including total dietary fiber (TDF, *n* = 22), cereal dietary fiber (CDF, *n* = 7), vegetable dietary fiber (VDF, *n* = 6), fruit dietary fiber (FDF, *n* = 5), legume dietary fiber (LDF, *n* = 4), SDF (*n* = 4), and IDF (*n* = 4). Figures 2–5 illustrate forest plots depicting associations between different levels of dietary fiber intake and specific cancer types. Figure 6 presents an overview of 13 dose-response analyses, detailing the connections between daily intake of various types of dietary fiber and multiple cancers. The detailed exclusion list is available in Supplementary Material Table S2.

## Main result

### Gastrointestinal cancers outcomes

#### Colorectal adenoma and cancer

**Fig. 7:**
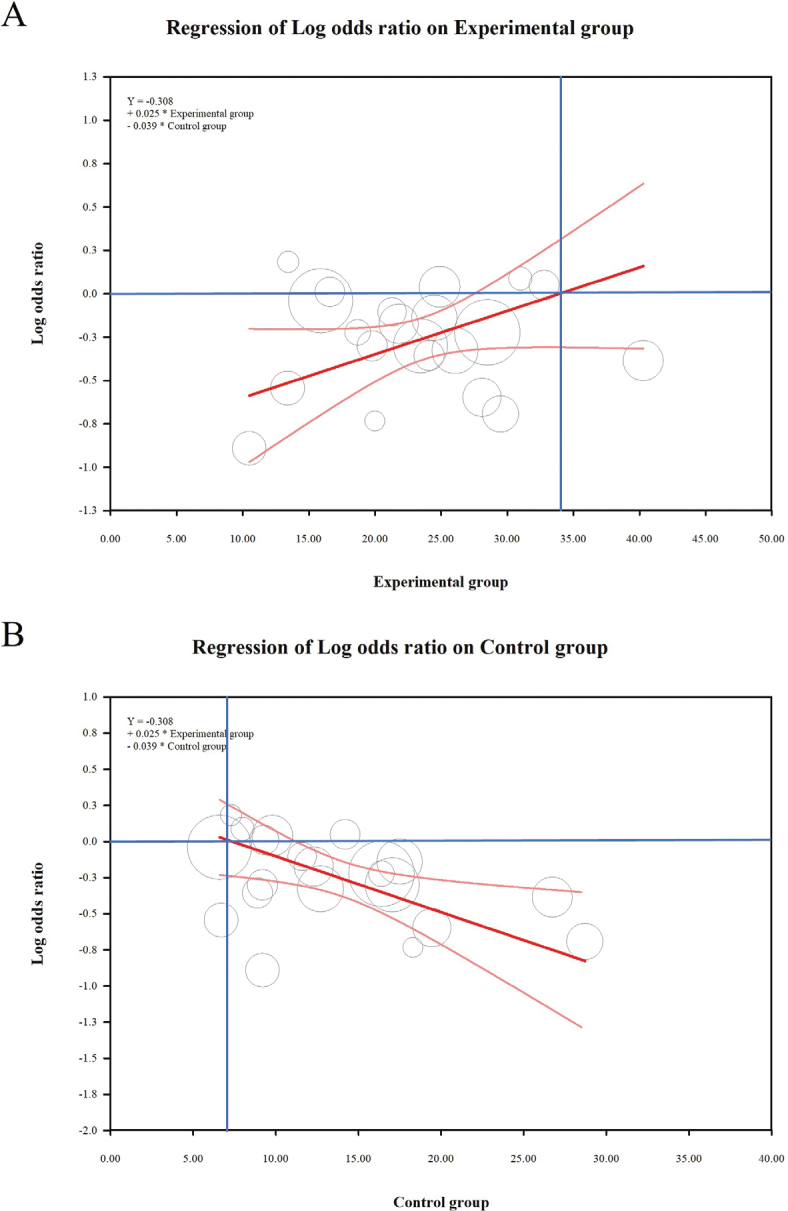
Meta-Regression Analysis of Dietary fiber intake and colon cancer incident rate. The X-axis represents the daily intake of dietary fiber (g/day), and the Y-axis is the Log odds ration of colon cancer incidence rate. (A) Experimental Group and (B) Control Group.

Dietary fiber reduces the incidence and recurrence of colorectal adenomas and cancers and reduces mortality and improves survival associated with colorectal cancer (Figs. 2 and 6). Ramezani’ team found that Insoluble fiber showed significant protection for the malignancy-related mortality (HR: 0.80, 95% CI: 0.73–0.88) (12). Nucci’s Team and Ben’s Team found that TDF intake reduced the risk of colorectal adenoma (OR: 0.71, 95% CI: 0.68 to 0.75) (13). The summary relative risks for cereal, vegetable, and FDFs were 0.97 (95% CI: 0.93–1.01), 0.93 (95% CI: 0.84–1.04), and 0.84 (95% CI: 0.76–0.94), respectively (14). High vs. low TDF intake was linked to a reduced incidence of colorectal cancer (RR: 0.88, 95% CI: 0.82–0.94); the RR for cereal, vegetable, legume, and FDFs was 0.90 (95% CI: 0.83–0.96), 0.98 (95% CI: 0.91–1.06), 0.89 (95% CI: 0.78–1.02), and 0.94 (95% CI: 0.85–1.04), respectively (15). Combining different types of dietary fiber, a 10 g/day increase in CDF resulted in a 3% higher recurrence rate (RR: 1.03, 95% CI: 0.62–1.71), while VDF showed a reduced recurrence rate (RR: 0.84, 95% CI: 0.71–0.98). A similar trend was observed for FDF (RR: 0.78, 95% CI: 0.65–0.93) (16). TDF increments of 10 g/day were linked to lower recurrence rates (RR: 0.90, 95% CI: 0.86–0.94). Specific types of fiber showed varied effects, with CDF (RR: 0.90, 95% CI: 0.83–0.97), VDF (RR: 0.98, 95% CI: 0.91–1.06), LDF (RR: 0.62, 95% CI: 0.27–1.42), SDF (RR: 0.78, 95% CI: 0.66–0.92), and IDF (RR: 0.77, 95% CI: 0.67–0.88) dietary fibers all exhibiting different impacts (3, 15). The highest dietary fiber consumption in colorectal cancer patients was linked to lower all-cause mortality (HR: 0.76, 95% CI: 0.65–0.88) and colorectal cancer-specific mortality (HR: 0.78, 95% CI: 0.63–0.97) (17). Gianfredi’s team reported that the RR for colon cancer was 0.74 (95% CI: 0.67–0.82), and for rectal cancer, it was 0.77 (95% CI: 0.66–0.89) (18, 19). A significant statistical association was found between TDF intake and the log odds of colon cancer outcomes (Supplementary Material Figure S1). The model demonstrated strong predictive power, with an explained variance (*R*²) of 0.75 (Supplementary Material Table S3). The meta-regression analysis yielded the equation: Y = −0.308 + 0.025 × Experimental group- 0.039 × Control group. For the experimental group, the log OR of colon cancer incidence rate increased slightly with higher daily fiber intake, indicating a positive correlation. Conversely, for the control group, the log OR decreased with higher daily intake, indicating an inverse relationship. The model suggests that the optimal range for preventing colon cancer occurrence is likely within 7–36 g/day (Fig. 7). This range is based on the balance between the positive correlation observed in the experimental group and the inverse relationship noted in the control group.

### Esophageal adenocarcinoma

Findings from meta-analyses suggest that increased consumption of dietary fiber markedly lowers the risk of Barrett’s esophagus (GERD) and ESCA. People consuming the highest levels of fiber exhibited a significant risk reduction compared to those with the lowest intake (RR: 0.52, 95% CI: 0.43–0.64) (20) (Fig. 2). GERD, a precancerous condition, is crucial in the progression of ESCA. Another analysis indicated that the greatest fiber consumption was linked to a lower risk of ESCA (HR: 0.50, 95% CI: 0.37–0.67) and ESCA (HR: 0.53, 95% CI: 0.31–0.90). A dose-response analysis revealed a 31% decrease in ESCA risk for each 10 g/day increase in fiber intake (RR: 0.69, 95% CI: 0.61–0.79) (20) (Fig. 6).

### Gastric cancer

Increased consumption of TDF substantially lowers the risk of developing gastric cancer. According to Zhang’s study, individuals with the highest TDF intake had a significantly lower risk of gastric cancer than those with the lowest intake (OR: 0.58, 95% CI: 0.49–0.67) (21) (Fig. 2). Furthermore, an analysis of the dose-response relationship indicated that each 10 g/day increment in dietary fiber intake led to a reduction in gastric cancer incidence (OR: 0.56, 95% CI: 0.45–0.71) (21) (Fig. 6).

### Pancreatic cancer

Mao’s study examined the impact of dietary fiber consumption on pancreatic cancer across 13 case-control studies and one cohort study. The findings revealed a strong inverse association between risk of pancreatic cancer and high fiber intake (OR: 0.52, 95% CI: 0.44–0.61) (22). Additionally, the dose-response analysis demonstrated that each 10 g/day increase in dietary fiber consumption was associated with a 12% reduction in pancreatic cancer risk (OR: 0.88, 95% CI: 0.84–0.92) (22) (Fig. 2).

### Liver cancer

Dietary fiber has been found to have an inverse relationship with the risk of liver cancer. According to Watling’s study, different types of liver cancer exhibited varying associations with TDF risk. Intrahepatic cholangiocarcinoma (ICC) was linked to an HR of 1.20 (95% CI: 064–2.24) and hepatocellular carcinoma (HCC) to an HR of 0.62 (95% CI: 0.45–0.86). Moreover, individuals with the highest intake of TDF experienced a 17% lower cancer mortality rate compared to those with the lowest intake (HR: 0.83, 95% CI: 0.69–1.01) (23). Furthermore, the dose-response analysis revealed that every 10 g/day increase in dietary fiber consumption was linked to a significant 17% decrease in the risk of developing liver cancer (HR: 0.83, 95% CI: 0.77–0.91) (23).

### Reproductive system cancers outcomes

#### Breast cancer outcomes

A meta-analysis showed that higher TDF intake is associated with a reduced breast cancer incidence rate (RR: 0.88, 95% CI: 0.83–0.93) (24) (Fig. 3). Analysis revealed a 4% reduction in breast cancer risk per additional 10 g/day of TDF (RR: 0.96, 95% CI: 0.92–0.98) (24) (Fig. 6). Moreover, each 10 g/day boost in CDF intake was linked to a 9% decrease in breast cancer risk (RR: 0.91, 95% CI: 0.79–1.04) (25) (Fig. 6). Huang’s research indicated that the highest fiber consumption was tied to a 37% reduction in breast cancer mortality from all causes (RR: 0.63, 95% CI: 0.52–0.77) and a 28% reduction in breast cancer-specific mortality (RR: 0.78, 95% CI: 0.54–0.96) (26) (Fig. 3). A detailed review of different fiber types showed varied risk reductions: CDF (RR: 0.97, 95% CI: 0.93–1.01), FDF (RR: 0.93, 95% CI: 0.89–0.96), VDF (RR: 0.95, 95% CI: 0.90–1.00), LDF (RR: 0.97, 95% CI: 0.92–1.03), SDF (RR: 0.90, 95% CI: 0.84–0.96), and IDF (RR: 0.93, 95% CI: 0.86–1.00) (27) (Fig. 3).

### Ovarian cancer

A meta-analysis of 567,742 participants and 8,200 ovarian cancer cases demonstrated that increased TDF consumption is linked to a decreased risk of ovarian cancer. Specifically, those consuming the most dietary fiber had a marked risk reduction relative to those with the lowest intake (RR: 0.70, 95% CI: 0.57–0.87) (28) (Fig. 3). Furthermore, the dose-response analysis showed that every additional 5 g/day of dietary fiber intake corresponded to a 3% decrease in ovarian cancer risk (RR: 0.97, 95% CI: 0.95–0.99) (28) (Fig. 6).

### Endometrial cancer

An analysis of 16 studies with 6,563 cases revealed that greater dietary fiber consumption is linked to a lower incidence of endometrial cancer (RR: 0.86, 95% CI: 0.78–0.93) (29) (Fig. 3). Another study by Kangning Chen’s group revealed that the highest intake of VDF, compared to the lowest, yielded an RR of 0.88 (95% CI: 0.68–1.13) for endometrial cancer (5). In contrast, the highest consumption of grain dietary fiber compared to the lowest was linked to an RR of 1.06 (95% CI: 0.58–1.32) for endometrial cancer (5) (Fig. 3).

### Prostate cancer

A comprehensive analysis of five cohort studies and 12 case-control studies, totaling 255,026 participants and 13,484 cases, investigated the relationship between dietary fiber consumption and prostate cancer. The results indicated an inverse relationship between high and low intake of TDF and the risk of prostate cancer (OR: 0.89, 95% CI: 0.77–1.01) (30) (Fig. 3).

## Genitourinary cancers outcomes

### Renal cell carcinoma

A review of 12 studies demonstrated that individuals with the highest intake of dietary fiber had a lower risk of RCC compared to those with the lowest intake (RR: 0.82, 95% CI: 0.72–0.92) (31) (Fig. 4). In a specific analysis by Tian-bao Huang, different types of dietary fiber showed varied associations with RCC risk. FDF was linked to an RR of 0.92 (95% CI: 0.80–0.92), VDF to an RR of 0.70 (95% CI: 0.49–1.00), grain fiber to an RR of 1.04 (95% CI: 0.91–1.18), and LDF to an RR of 0.80 (95% CI: 0.69–0.93) (32) (Fig. 4). Furthermore, increasing LDF intake by 2.5 g/day corresponded to a 12% decrease in RCC risk (RR: 0.88, 95% CI: 0.61–1.25) (32) (Fig. 6).

### Bladder cancer

An analysis covering 574,726 participants and 3,214 cases investigated the connection between dietary fiber intake and bladder cancer. Results showed that the highest TDF consumption was tied to a lower incidence of bladder cancer compared to the lowest consumption (33) (Fig. 4). Furthermore, dose-response analysis indicated that every additional 5 g/day of dietary fiber intake corresponded to a decreased risk of bladder cancer (HR: 0.96, 95% CI: 0.94–0.98) (33) (Fig. 6).

### All cancer mortality outcomes

Yao’s study found that the inverse correlation between cancer mortality was specifically observed for TDF (RR: 0.82, 95% CI: 0.77–0.87) and CDF (RR: 0.86, 95% CI: 0.83–0.90), whereas the current study did not reveal any significant connection between the intake of insoluble or soluble fiber and cancer mortality (RR: 0.92, 95% CI: 0.74–1.14) and (RR: 0.97, 95% CI: 0.63–1.51) (34), respectively. Additional findings showed an inverse relationship between cereal fiber intake and all-cause mortality risk (RR: 0.81, 95% CI: 0.79–0.83) (35) (Fig. 5). The dose-response analysis demonstrated that for every 10 g/day increase in TDF intake, cancer mortality decreased by 9% (RR: 0.91, 95% CI: 0.88–0.93) (34) (Fig. 6).

## Evaluation of systematic review quality

The AMSTAR-2 scale was employed to assess the quality of the 24 included studies. One study (4%) on colorectal cancer was rated ‘high’ quality, one study (4%) on pancreatic cancer was rated ‘medium’ quality, three studies (12%) received ‘low’ quality ratings, and 19 studies (80%) were categorized as ‘very low’ quality. Detailed evaluation results are available in Supplementary Material Table S4. The AMSTAR-2 tool, a widely recognized instrument, was used to evaluate the quality of systematic reviews and meta-analyses, focusing on 16 criteria with an emphasis on seven key areas: question formulation, study inclusion, study exclusion, risk of bias, outcome bias, reporting bias, and other biases. Many studies did not adequately describe the selection of included study types and lacked lists of excluded studies with justifications for their exclusion. Figures 2–6 display the AMSTAR-2 scores for the 24 meta-analyses.

### Heterogeneity

Heterogeneity among the included meta-analyses was substantial, with approximately 60% of cancer outcomes exhibiting significant heterogeneity (I2 > 50% or *P*-value from Cochran’s Q test < 0.1). We identified several sources of heterogeneity, including differences in study settings, geographic regions, ethnicities, participant age, sex, study quality, and sample sizes as well as variations in follow-up duration and the extent of confounding adjustments. For example, studies analyzing colorectal cancer outcomes showed marked variation due to differences in dietary fiber sources (e.g. cereal vs. vegetable fiber) and populations from Western versus Asian countries. This heterogeneity influences the overall reliability of our conclusions, particularly where the variability across studies was higher, suggesting that results should be interpreted with caution. Meta-regression analysis was employed to investigate effect modification, and while certain factors such as fiber type were significant, residual heterogeneity persisted, indicating the need for further research to clarify the optimal levels of dietary fiber intake.

## Discussion

### Main findings and possible explanations

The quality of the included meta-analyses was systematically assessed using the AMSTAR-2 tool, revealing that the majority of studies (80%) were rated as ‘very low’ quality, primarily due to methodological flaws such as inadequate handling of heterogeneity and insufficiently reported risk of bias. Only one study was rated ‘high’ quality, and this was focused on colorectal cancer outcomes. The low methodological quality of most studies suggests that bias – especially publication bias and selection bias – may have influenced the results, potentially overstating the protective effect of dietary fiber in some cancer types. For instance, studies that did not adequately account for confounders such as baseline dietary habits or lifestyle factors may have introduced bias, complicating the interpretation of results for cancers such as breast and endometrial cancers. Given the suboptimal quality of the evidence base, conclusions regarding the protective effects of dietary fiber should be approached with caution, and future high-quality systematic reviews and meta-analyses are necessary to strengthen the evidence. Therefore, due caution should be exercised when interpreting the significant associations observed between dietary fiber consumption and certain cancer risks. Additionally, the study findings showed that individuals with higher dietary fiber intake were at a lower risk of several types of cancer compared to those with lower fiber intake, suggesting that dietary fiber may possess a certain degree of cancer-preventive efficacy.

Our study demonstrated that higher dietary fiber consumption is associated with reduced incidence, mortality, and recurrence rates of multiple cancers. These include gastrointestinal cancers such as esophageal adenocarcinoma, stomach cancer, and colorectal cancer (3, 12,–15, 15–21); liver cancer (23); reproductive system cancers like breast, ovarian, and endometrial cancers (5, 24, 26–29); and urinary system cancers, including prostate cancer, RCC, and bladder cancer (22, 30–33). Additionally, a notable decrease in overall cancer mortality was observed (34, 35). It is particularly emphasized that we have found the optimal dietary fiber intake for preventing colon cancer occurrence is most likely within 7–36 g/day.

Consistent with the previous research, our study reinforced the connection between elevated fiber consumption and a lower likelihood of various illnesses, particularly those affecting the digestive, reproductive, and urinary systems. This aligns with the findings of Andrew Reynolds’ team, who, through a rigorous examination of 185 prospective studies and 58 clinical trials, underscored the protective role of increased fiber intake in guarding against cancers linked to the gastrointestinal and reproductive systems (36). Moreover, our results on genitourinary cancers are consistent with a significant long-term cohort study involving 491,841 American male and female participants, which found that higher intake of dietary fiber and fiber-dense plant foods was significantly linked to a decreased risk of RCC (37).

Our research consistently shows that dietary fiber is associated with lower incidence, recurrence, and mortality rates of colorectal cancer, leading to positive outcomes. This protective effect is attributed to fiber’s multifaceted functions, including its capacity to physically expel carcinogens from the digestive system, promoting cell elimination, and modulating the gut environment for a healthier mucosal barrier (38). The transformation of fiber into short-chain fatty acids like butyrate, acetate, and propionate amplifies its protective benefits by fortifying gut barrier integrity, triggering apoptosis in cancer cells, and suppressing tumor growth (39, 40). Additionally, fiber mitigates gut inflammation, a key driver of carcinogenesis, by regulating cell proliferation (2, 41). Beyond the gastrointestinal tract, fiber enhances gut microbiota diversity and functionality, enhancing overall gut barrier function and reducing systemic inflammation (42, 43). Fiber increases stool volume and speeds up transit time, thereby reducing the gut lining’s exposure to potential carcinogens. This mechanism also applies to other gastrointestinal cancers, such as stomach and ESCA (39). Fiber’s impact on pancreatic cancer is less studied, but preliminary evidence suggests a similar protective mechanism (44). Further investigation is necessary to fully elucidate the scope of fiber’s protective effects against various gastrointestinal cancers.

Dietary fiber is essential in preventing reproductive system cancers by modulating hormone levels and improving insulin sensitivity, both of which are critical for reducing cancer risk. Epidemiological studies show a strong link between high fiber consumption and decreased breast cancer mortality, attributed to fiber’s capacity to reduce circulating estrogen levels (27). Fiber binds to estrogen in the colon, enhancing its excretion and decreasing reabsorption, while also inhibiting β-glucuronidase activity, which hydrolyzes conjugated estrogens (45, 46). This reduction in estrogen availability significantly reduces breast cancer development. Studies suggest a connection between increased fiber consumption and a reduced likelihood of ovarian cancer by lowering blood estrogen concentrations and enhancing the protective effects of lignans and phytoestrogens (47). Fiber’s impact on glycemic control and insulin sensitivity aids in regulating insulin-like growth factor 1, which, when elevated, promotes cell proliferation and inhibits apoptosis, contributing to ovarian cancer risk (48). For endometrial cancer prevention, consuming dietary fiber is crucial for weight control since obesity heightens the risk by affecting hormone and growth factor levels. Fiber aids in regulating body fat and managing insulin resistance, particularly in abdominal obesity, thereby reducing hyperinsulinemia and lowering the risk of endometrial cancer (49). Furthermore, fiber’s interaction with bile acids contributes to cancer prevention by reducing reabsorption in the liver, leading to increased cholesterol utilization for bile acid production and subsequent plasma cholesterol decrease (50). This consequently lowers the likelihood of hormone-driven malignancies, including endometrial carcinoma, highlighting the role of fiber in a preventive diet (51).

Our study found that dietary fiber intake positively influenced the incidence of urinary tract cancers. Recent studies indicate that an elevated consumption of dietary fiber may exhibit a preventive role in certain urogenital cancers, such as prostate, kidney, and bladder, through its regulatory effects on hormones and inflammation. Fiber’s protective mechanism is multifaceted, particularly in prostate cancer, where it may lower the risk by modulating hormonal balance. Lignans found in fiber-rich foods, for instance, have been associated with a decreased risk due to their influence on sex hormone-binding globulin, which governs hormone activity linked to cancer development (30, 52). In kidney cancer, specifically RCC, dietary fiber’s impact lies in its capacity to stabilize blood sugar levels, preventing hyperglycemia, a factor that can fuel cancer growth. Vegetables and legumes, rich in fiber, contribute significantly to this protection by regulating post-meal glucose and insulin responses (37, 53). Bladder cancer incidence may also be reduced by dietary fiber, as it helps lower the glycemic load, thereby mitigating the carcinogenic effects of hyperglycemia and hyperinsulinemia (54, 55). While some studies yield inconclusive results, the general trend suggests a potential protective effect of fiber intake in cancer prevention. Therefore, incorporating fiber-rich foods into a comprehensive cancer prevention strategy is of paramount importance.

However, there were some unexpected findings in our study. Our research suggests a potential positive correlation between the consumption of grain fiber, FDF, and IDF and the incidence of colorectal cancer. The influence of IDF on gut flora metabolism and carcinogen binding varies across different sites of colorectal malignancy, affecting cancer occurrence rates, as per studies (56, 57). Future research should further investigate the role of fiber solubility in confirming this uneven effect. Additionally, inconsistent findings were observed, particularly about the influence of grain fiber on uterine and renal cell cancer risks. Chen’s research highlighted inconsistencies linking grain fiber consumption and endometrial cancer risk. This discrepancy might arise because grain fiber is not considered an isolated nutrient within the study; elevated grain fiber intake could indicate increased carbohydrate consumption, which raises the likelihood of obesity and uterine cancer (58–60). Factors affecting Tian-bao Huang’s study include variations in Food Frequency Questionnaire items, differences in geographical locations, study design variations, and adjustments for confounding factors. These elements might explain the unexpected correlation between grain fiber consumption and the risk of RCC.

### Advantages and drawbacks of the research

The present research provided an in-depth analysis of this relationship between fiber consumption and cancer outcomes, emphasizing incidence, mortality, and recurrence rates across various cancer types. By conducting separate analyses for different fiber types, we were able to discern their potential impact on cancer risk, thereby informing targeted nutritional recommendations and intervention strategies. The dose-response evaluation in our study quantified the correlation of fiber consumption with cancer likelihood, providing valuable insights into the extent to which dietary fiber affects health. We prioritized studies with large sample sizes and excluded redundant data to minimize bias, enhancing the reliability of our findings.

Although our study has significant methodological strengths, it also has limitations. There is a possibility that incomplete data retrieval from the databases included in our review resulted in the omission of pertinent studies. Another potential scenario is that only the outcomes related to colorectal cancer exhibit statistical significance, whereas the meta-regression analyses of other outcomes fail to meet expectations, thereby limiting the comprehensiveness of the manuscript. Furthermore, our study comprised 26 meta-analyses, all based on observational studies, and did not address the challenges inherent in cohort and case-control research, such as recall and selection biases, which may affect the reliability of the findings.

## Conclusions

The results of our study support public health initiatives that highlight the importance of dietary fiber in reducing cancer risk and offer proof of a notable link between fiber consumption and colorectal cancer incidence. These findings guide the creation of public health strategies and personalized nutrition recommendations. However, our research primarily encompasses studies from Europe and North America, with a limited number from Asia and other regions. Compared to other areas, there is a clear relationship between Western diets (rich in fats, sugars, and animal products) and an increased risk of colorectal cancer and ovarian cancer, among others (61). Consequently, there may be significant geographical variations in our findings. For instance, Vincenza Gianfredi’s research into the effects of dietary fiber on colorectal cancer reveals a higher negative correlation in Western countries compared to Asia (19). A study conducted in North America also found a significant negative correlation between total fiber intake and the risk of ovarian cancer (28). Therefore, we believe that reducing the intake of high-sugar, high-fat diets by residents of Western countries may lower the risk of various cancers. In addition, studies by Jing Zhao (17), Daniele Nucci (13), and others have found that fiber intake has a higher protective effect in male colorectal cancer patients when analyzed by gender. The possible reason for this result is the gender difference in dietary habits, as previous research has shown that men typically consume less dietary fiber than women. Therefore, it is recommended that male colorectal cancer patients should increase their intake of dietary fiber to resist cancer. In addition, Sumei Chen (24) has indicated that the intake of dietary fiber is significantly related to a reduced risk of developing breast cancer, especially in postmenopausal women. Although no connection was observed in pre-menopausal women, the protective effect will also increase with an increase in dietary fiber intake (24). Additionally, studies have shown that soluble fiber is consistent in its effect on breast cancer risk in both pre- and post-menopausal women (26). Due to significant geographical variations, which largely contribute to heterogeneity, the evidence we have collected is limited, and further research is needed into the factors affecting breast cancer risk within dietary fiber. Furthermore, the European Prospective Investigation into Cancer and Nutrition (EPIC) reported that as fiber intake increases, the risk of colorectal cancer decreases linearly (15). However, a study conducted in Utah, USA, on White CRC patients found that higher fiber intake was associated with the opposite of CRC survival rates. Due to their lifestyle that prohibits alcohol, coffee, or tobacco, this unique population characteristic may affect the comparability and generalizability of the study results (17). Therefore, understanding these differences in geography, diet, and lifestyle can help narrow the differences in research results, reduce the cancer burden on different populations, and provide effective information for public health interventions.

## Supplementary Material



## Data Availability

The data derived from the systematic reviews incorporated within this umbrella review are sourced from the respective original publications, as cited in the References section.

## References

[1] Wang S, Zhang J, Li J, Wang J, Liu W, Zhang Z, et al. Label-free quantitative proteomics reveals the potential mechanisms of insoluble dietary fiber from okara in improving hepatic lipid metabolism of high-fat diet-induced mice. J Proteomics 2023; 287: 104980. doi: 10.1016/j.jprot.2023.104980 37499746

[2] Barber TM, Kabisch S, Pfeiffer AFH, Weickert MO. The health benefits of dietary fibre. Nutrients 2020; 12(10): 3209. doi: 10.3390/nu12103209 33096647 PMC7589116

[3] Arayici ME, Mert-Ozupek N, Yalcin F, Basbinar Y, Ellidokuz H. Soluble and insoluble dietary fiber consumption and colorectal cancer risk: a systematic review and meta-analysis. Nutr Cancer 2022; 74(7): 2412–25. doi: 10.1080/01635581.2021.2008990 34854791

[4] Xu K, Sun Q, Shi Z, Zou Y, Jiang X, Wang Y, et al. A dose-response meta-analysis of dietary fiber intake and breast cancer risk. Asia Pac J Public Health 2022; 34(4): 331–7. doi: 10.1177/10105395211072997 35073762

[5] Chen K, Zhao Q, Li X, Zhao J, Li P, Lin S, et al. Dietary fiber intake and endometrial cancer risk: a systematic review and meta-analysis. Nutrients 2018; 10(7): 945. doi: 10.3390/nu10070945 30037138 PMC6073518

[6] Nutrition, Food Labeling, and Critical Foods. Health claim notification for whole grain foods. FDA; 2022. Available from: https://www.fda.gov/food/food-labeling-nutrition/health-claim-notification-whole-grain-foods [cited 2 March 2024].

[7] Katagiri R, Goto A, Shimazu T, Yamaji T, Sawada N, Iwasaki M, et al. Dietary fiber intake and risk of gastric cancer: the Japan Public Health Center-based prospective study. Int J Cancer 2021; 148(11): 2664–73. doi: 10.1002/ijc.33450 33348433

[8] Kim H, Youn J, Yang SY, Song JH, Kim YS, Lee JE. Association between dietary fiber intake and colorectal adenoma. Nutr Cancer 2022; 74(10): 3446–56. doi: 10.1080/01635581.2022.2083189 35658767

[9] Luo J, Xu X. Dietary fiber intake and the risk of bladder cancer in the Prostate, Lung, Colorectal and Ovarian (PLCO) cohort. Carcinogenesis 2020; 41(4): 478–82. doi: 10.1093/carcin/bgz187 31872237 PMC7298621

[10] Singh V, Yeoh BS, Chassaing B, Xiao X, Saha P, Aguilera Olvera R, et al. Dysregulated microbial fermentation of soluble fiber induces cholestatic liver cancer. Cell 2018; 175(3): 679–94.e22. doi: 10.1016/j.cell.2018.09.004 30340040 PMC6232850

[11] Guyatt G, Oxman AD, Akl EA, Kunz R, Vist G, Brozek J, et al. GRADE guidelines: 1. Introduction-GRADE evidence profiles and summary of findings tables. J Clin Epidemiol 2011; 64(4): 383–94. doi: 10.1016/j.jclinepi.2010.04.026 21195583

[12] Ramezani F, Pourghazi F, Eslami M, Gholami M, Mohammadian Khonsari N, Ejtahed H-S, et al. Dietary fiber intake and all-cause and cause-specific mortality: an updated systematic review and meta-analysis of prospective cohort studies. Clin Nutr 2024; 43(1): 65–83. doi: 10.1016/j.clnu.2023.11.005 38011755

[13] Nucci D, Fatigoni C, Salvatori T, Nardi M, Realdon S, Gianfredi V. Association between dietary fibre intake and colorectal adenoma: a systematic review and meta-analysis. Int J Environ Res Public Health 2021; 18(8): 4168. doi: 10.3390/ijerph18084168 33920845 PMC8071151

[14] Ben Q, Sun Y, Chai R, Qian A, Xu B, Yuan Y. Dietary fiber intake reduces risk for colorectal adenoma: a meta-analysis. Gastroenterology 2014; 146(3): 689–99.e6. doi: 10.1053/j.gastro.2013.11.003 24216326

[15] Aune D, Chan DSM, Lau R, Vieira R, Greenwood DC, Kampman E, et al. Dietary fibre, whole grains, and risk of colorectal cancer: systematic review and dose-response meta-analysis of prospective studies. BMJ 2011; 343: d6617. doi: 10.1136/bmj.d6617 22074852 PMC3213242

[16] Oh H, Kim H, Lee DH, Lee A, Giovannucci EL, Kang S-S, et al. Different dietary fibre sources and risks of colorectal cancer and adenoma: a dose–response meta-analysis of prospective studies. Br J Nutr 2019; 122(6): 605–15. doi: 10.1017/S0007114519001454 31495339

[17] Zhao J, Zhu Y, Du M, Wang Y, Vallis J, Parfrey PS, et al. Association between dietary fiber intake and mortality among colorectal cancer survivors: results from the newfoundland familial colorectal cancer cohort study and a meta-analysis of prospective studies. Cancers (Basel) 2022; 14(15): 3801. doi: 10.3390/cancers14153801 35954465 PMC9367345

[18] Gianfredi V, Salvatori T, Villarini M, Moretti M, Nucci D, Realdon S. Is dietary fibre truly protective against colon cancer? A systematic review and meta-analysis. Int J Food Sci Nutr 2018; 69(8): 904–15. doi: 10.1080/09637486.2018.1446917 29516760

[19] Gianfredi V, Nucci D, Salvatori T, Dallagiacoma G, Fatigoni C, Moretti M, et al. Rectal cancer: 20% risk reduction thanks to dietary fibre intake. systematic review and meta-analysis. Nutrients 2019; 11(7): 1579. doi: 10.3390/nu11071579 31336939 PMC6683071

[20] Sun L, Zhang Z, Xu J, Xu G, Liu X. Dietary fiber intake reduces risk for Barrett’s esophagus and esophageal cancer. Crit Rev Food Sci Nutr 2017; 57(13): 2749–57. doi: 10.1080/10408398.2015.1067596 26462851

[21] Zhang Z, Xu G, Ma M, Yang J, Liu X. Dietary fiber intake reduces risk for gastric cancer: a meta-analysis. Gastroenterology 2013; 145(1): 113–20.e3. doi: 10.1053/j.gastro.2013.04.001 23567349

[22] Mao Q-Q, Lin Y-W, Chen H, Qin J, Zheng X-Y, Xu X, et al. Dietary fiber intake is inversely associated with risk of pancreatic cancer: a meta-analysis. Asia Pac J Clin Nutr 2017; 26(1): 89–96. doi: 10.6133/apjcn.102015.03 28049267

[23] Watling CZ, Wojt A, Florio AA, Butera G, Albanes D, Weinstein SJ, et al. Fiber and whole grain intakes in relation to liver cancer risk: an analysis in 2 prospective cohorts and systematic review and meta-analysis of prospective studies. Hepatology 2024; 80(3): 552–65. doi: 10.1097/HEP.0000000000000819 38441973 PMC11803500

[24] Chen S, Chen Y, Ma S, Zheng R, Zhao P, Zhang L, et al. Dietary fibre intake and risk of breast cancer: a systematic review and meta-analysis of epidemiological studies. Oncotarget 2016; 7(49): 80980–9. doi: 10.18632/oncotarget.13140 27829237 PMC5348370

[25] Aune D, Chan DSM, Greenwood DC, Vieira AR, Rosenblatt DAN, Vieira R, et al. Dietary fiber and breast cancer risk: a systematic review and meta-analysis of prospective studies. Ann Oncol 2012; 23(6): 1394–402. doi: 10.1093/annonc/mdr589 22234738

[26] Jayedi A, Emadi A, Khan TA, Abdolshahi A, Shab-Bidar S. Dietary fiber and survival in women with breast cancer: a dose-response meta-analysis of prospective cohort studies. Nutr Cancer 2021; 73(9): 1570–80. doi: 10.1080/01635581.2020.1803928 32795218

[27] Farvid MS, Spence ND, Holmes MD, Barnett JB. Fiber consumption and breast cancer incidence: a systematic review and meta-analysis of prospective studies. Cancer 2020; 126(13): 3061–75. doi: 10.1002/cncr.32816 32249416

[28] Xu H, Ding Y, Xin X, Wang W, Zhang D. Dietary fiber intake is associated with a reduced risk of ovarian cancer: a dose-response meta-analysis. Nutr Res 2018; 57: 1–11. doi: 10.1016/j.nutres.2018.04.011 30122191

[29] Li H, Mao H, Yu Y, Nan Y. Association between dietary fiber and endometrial cancer: a meta-analysis. Nutr Cancer 2020; 72(6): 959–67. doi: 10.1080/01635581.2019.1670218 31584301

[30] Sheng T, Shen R, Shao H, Ma T. No association between fiber intake and prostate cancer risk: a meta-analysis of epidemiological studies. World J Surg Oncol 2015; 13: 264. doi: 10.1186/s12957-015-0681-8 26315558 PMC4552444

[31] Xu X, Zhu Y, Li J, Wang S. Dietary fiber, glycemic index, glycemic load and renal cell carcinoma risk. Carcinogenesis 2019; 40(3): 441–7. doi: 10.1093/carcin/bgz049 30859214

[32] Huang T, Ding P, Chen J, Yan Y, Zhang L, Liu H, et al. Dietary fiber intake and risk of renal cell carcinoma: evidence from a meta-analysis. Med Oncol 2014; 31(8): 125. doi: 10.1007/s12032-014-0125-2 25038944

[33] Yu EYW, Wesselius A, Mehrkanoon S, Brinkman M, van den Brandt P, White E, et al. Grain and dietary fiber intake and bladder cancer risk: a pooled analysis of prospective cohort studies. Am J Clin Nutr 2020; 112(5): 1252–66. doi: 10.1093/ajcn/nqaa215 32778880 PMC7657329

[34] Yao F, Ma J, Cui Y, Huang C, Lu R, Hu F, et al. Dietary intake of total vegetable, fruit, cereal, soluble and insoluble fiber and risk of all-cause, cardiovascular, and cancer mortality: systematic review and dose-response meta-analysis of prospective cohort studies. Front Nutr 2023; 10: 1153165. doi: 10.3389/fnut.2023.1153165 37854351 PMC10579821

[35] Hajishafiee M, Saneei P, Benisi-Kohansal S, Esmaillzadeh A. Cereal fibre intake and risk of mortality from all causes, CVD, cancer and inflammatory diseases: a systematic review and meta-analysis of prospective cohort studies. Br J Nutr 2016; 116(2): 343–52. doi: 10.1017/S0007114516001938 27193606

[36] Reynolds A, Mann J, Cummings J, Winter N, Mete E, Te Morenga L. Carbohydrate quality and human health: a series of systematic reviews and meta-analyses. Lancet 2019; 393(10170): 434–45. doi: 10.1016/S0140-6736(18)31809-9 30638909

[37] Daniel CR, Park Y, Chow W-H, Graubard BI, Hollenbeck AR, Sinha R. Intake of fiber and fiber-rich plant foods is associated with a lower risk of renal cell carcinoma in a large US cohort. Am J Clin Nutr 2013; 97(5): 1036–43. doi: 10.3945/ajcn.112.045351 23515007 PMC3628376

[38] Lahmann PH, Ibiebele TI, Webb PM, Nagle CM, Whiteman DC. A case-control study of glycemic index, glycemic load and dietary fiber intake and risk of adenocarcinomas and squamous cell carcinomas of the esophagus: the Australian Cancer Study. BMC Cancer 2014; 14: 877. doi: 10.1186/1471-2407-14-877 25421419 PMC4255966

[39] Song M, Wu K, Meyerhardt JA, Ogino S, Wang M, Fuchs CS, et al. Fiber intake and survival after colorectal cancer diagnosis. JAMA Oncol 2018; 4(1): 71–9. doi: 10.1001/jamaoncol.2017.3684 29098294 PMC5776713

[40] Bultman SJ. Molecular pathways: gene-environment interactions regulating dietary fiber induction of proliferation and apoptosis via butyrate for cancer prevention. Clin Cancer Res 2014; 20(4): 799–803. doi: 10.1158/1078-0432.CCR-13-2483 24270685 PMC3944646

[41] Singh N, Baby D, Rajguru JP, Patil PB, Thakkannavar SS, Pujari VB. Inflammation and cancer. Ann Afr Med 2019; 18(3): 121–6. doi: 10.4103/aam.aam_56_18 31417011 PMC6704802

[42] O’Keefe SJD. Diet, microorganisms and their metabolites, and colon cancer. Nat Rev Gastroenterol Hepatol 2016; 13(12): 691–706. doi: 10.1038/nrgastro.2016.165 27848961 PMC6312102

[43] De Vries J. Effects of cereal fiber on bowel function: a systematic review of intervention trials. WJG 2015; 21(29): 8952. doi: 10.3748/wjg.v21.i29.8952 26269686 PMC4528039

[44] Hu J-X, Zhao C-F, Chen W-B, Liu Q-C, Li Q-W, Lin Y-Y, et al. Pancreatic cancer: a review of epidemiology, trend, and risk factors. World J Gastroenterol 2021; 27(27): 4298–321. doi: 10.3748/wjg.v27.i27.4298 34366606 PMC8316912

[45] Zengul AG, Demark-Wahnefried W, Barnes S, Morrow CD, Bertrand B, Berryhill TF, et al. Associations between dietary fiber, the fecal microbiota and estrogen metabolism in postmenopausal women with breast cancer. Nutr Cancer 2021; 73(7): 1108–17. doi: 10.1080/01635581.2020.1784444 32590914 PMC7875566

[46] Flores R, Shi J, Fuhrman B, Xu X, Veenstra TD, Gail MH, et al. Fecal microbial determinants of fecal and systemic estrogens and estrogen metabolites: a cross-sectional study. J Transl Med 2012; 10: 253. doi: 10.1186/1479-5876-10-253 23259758 PMC3552825

[47] Jeon S-Y, Hwang K-A, Choi K-C. Effect of steroid hormones, estrogen and progesterone, on epithelial mesenchymal transition in ovarian cancer development. J Steroid Biochem Mol Biol 2016; 158: 1–8. doi: 10.1016/j.jsbmb.2016.02.005 26873134

[48] Nagle CM, Kolahdooz F, Ibiebele TI, Olsen CM, Lahmann PH, Green AC, et al. Carbohydrate intake, glycemic load, glycemic index, and risk of ovarian cancer. Ann Oncol 2011; 22(6): 1332–8. doi: 10.1093/annonc/mdq595 21131370

[49] Yasin HK, Taylor AH, Ayakannu T. A narrative review of the role of diet and lifestyle factors in the development and prevention of endometrial cancer. Cancers 2021; 13(9): 2149. doi: 10.3390/cancers13092149 33946913 PMC8125712

[50] Ocvirk S, O’Keefe SJD. Dietary fat, bile acid metabolism and colorectal cancer. Semin Cancer Biol 2021; 73: 347–55. doi: 10.1016/j.semcancer.2020.10.003 33069873

[51] He J, Siu MKY, Ngan HYS, Chan KKL. Aberrant cholesterol metabolism in ovarian cancer: identification of novel therapeutic targets. Front Oncol 2021; 11: 738177. doi: 10.3389/fonc.2021.738177 34820325 PMC8606538

[52] Sawada N, Iwasaki M, Yamaji T, Shimazu T, Sasazuki S, Inoue M, et al. Fiber intake and risk of subsequent prostate cancer in Japanese men. Am J Clin Nutr 2015; 101(1): 118–25. doi: 10.3945/ajcn.114.089581 25527755

[53] Solarek W, Czarnecka AM, Escudier B, Bielecka ZF, Lian F, Szczylik C. Insulin and IGFs in renal cancer risk and progression. Endocr Relat Cancer 2015; 22(5): R253–64. doi: 10.1530/ERC-15-0135 26330483

[54] Evan YY, Wesselius A, Mehrkanoon S, Brinkman M, Van Den Brandt P, White E, et al. Grain and dietary fiber intake and bladder cancer risk: a pooled analysis of prospective cohort studies. Am J Clin Nutr 2020; 112(5): 1252–66. doi: 10.1093/ajcn/nqaa215 32778880 PMC7657329

[55] Augustin LSA, Taborelli M, Montella M, Libra M, La Vecchia C, Tavani A, et al. Associations of dietary carbohydrates, glycaemic index and glycaemic load with risk of bladder cancer: a case–control study. Br J Nutr 2017; 118(9): 722–9. doi: 10.1017/S0007114517002574 28990544

[56] Holscher HD. Dietary fiber and prebiotics and the gastrointestinal microbiota. Gut Microbes 2017; 8(2): 172–84. doi: 10.1080/19490976.2017.1290756 28165863 PMC5390821

[57] Koropatkin NM, Cameron EA, Martens EC. How glycan metabolism shapes the human gut microbiota. Nat Rev Microbiol 2012; 10(5): 323–35. doi: 10.1038/nrmicro2746 22491358 PMC4005082

[58] Jain MG, Rohan TE, Howe GR, Miller AB. A cohort study of nutritional factors and endometrial cancer. Eur J Epidemiol 2000; 16(10): 899–905. doi: 10.1023/a:1011012621990 11338120

[59] Cui X, Rosner B, Willett WC, Hankinson SE. Dietary fat, fiber, and carbohydrate intake in relation to risk of endometrial cancer. Cancer Epidemiol Biomarkers Prev 2011; 20(5): 978–89. doi: 10.1158/1055-9965.EPI-10-1089 21393567 PMC3089715

[60] Aarestrup J, Kyrø C, Christensen J, Kristensen M, Würtz AML, Johnsen NF, et al. Whole grain, dietary fiber, and incidence of endometrial cancer in a Danish cohort study. Nutr Cancer 2012; 64(8): 1160–8. doi: 10.1080/01635581.2012.723786 23163844

[61] Miller KD, Nogueira L, Mariotto AB, et al. Cancer treatment and survivorship statistics, 2019. CA Cancer J Clin 2019; 69(5): 363–85. doi: 10.3322/caac.21565 31184787

