# Supplementary material for: Dietary fiber consumption and outcomes of different cancers: an umbrella review
Source: Food Nutr Res. 2025 Jan 3;69:10.29219/fnr.v69.11034. doi: 10.29219/fnr.v69.11034 (PMC12509222; doi:10.29219/fnr.v69.11034)
Supplement: Supplementary file 1 [file FNR-69-11034-s1.docx]

**Supplementary Table S1.** Search strategy

Embase search strategy (via OvidSP) From 1974 to July 20, 2023

| 1. dietary fiber.mp. or exp *dietary fiber/  2. dietary fiber.ab,kf,kw,sh,ti.  3. "vegetarian*".ab,kf,kw,sh,ti.  4. "vegan*".ab,kf,kw,sh,ti.  5. macrobiotic.ab,kf,kw,sh,ti.  6. food fiber.ab,kf,kw,sh,ti.  7. whole grain.ab,kf,kw,sh,ti.  8. fruit.ab,kf,kw,sh,ti.  9. wheat bran.ab,kf,kw,sh,ti.  10. wheat brans.ab,kf,kw,sh,ti.  11. roughage.ab,kf,kw,sh,ti.  12. roughages.ab,kf,kw,sh,ti.  13. grain.ab,kf,kw,sh,ti.  14. grains.ab,kf,kw,sh,ti.  15. bread.ab,kf,kw,sh,ti.  16. bread.mp. or exp *bread/  17. exp *fruit/ or exp *fruit vegetable/ or Fruit.mp.  18. polysaccharide.ab,kf,kw,sh,ti.  19. polysaccharides.ab,kf,kw,sh,ti.  20. exp *vegetable/ or Vegetable.mp.  21. cereal.ab,kf,kw,sh,ti.  22. cereals.ab,kf,kw,sh,ti.  23. 1 or 2 or 3 or 4 or 5 or 6 or 7 or 8 or 9 or 10 or 11 or 12 or 13 or 14 or 15 or 16 or 17 or 18 or 19 or 20 or 21 or 22  24. tumor.ab,kf,kw,sh,ti.  25. Cancer.ab,kf,kw,sh,ti.  26. Cancer.mp. or exp *malignant neoplasm/  27. tumor.mp. or exp *neoplasm/  28. "carcinoma*".ab,kf,kw,sh,ti.  29. neoplasm.ab,kf,kw,sh,ti.  30. "tumo*".ab,kf,kw,sh,ti.  31. 24 or 25 or 26 or 27 or 28 or 29 or 30  32. 23 and 31  33. meta-analysis.mp. or exp *meta analysis/  34. exp *"review"/ or systematic review.mp. or exp *"systematic review"/  35. pooled analysis.ab,kf,kw,sh,ti.  36. meta-analyzed.ab,kf,kw,sh,ti.  37. meta-analysis.ab,kf,kw,sh,ti.  38. systematic review.ab,kf,kw,sh,ti.  39. 33 or 34 or 35 or 36 or 37 or 38  40. 32 and 39 |
| --- |

**Supplementary Table S2.** Literature screening details and exclusion reasons

| **Excluded article** | **Reasons for exclusion** |
| --- | --- |
|  |  |
| Liu L, Wang S, Liu J. Fiber consumption and all-cause, cardiovascular, and cancer mortalities: a systematic review and meta-analysis of cohort studies. Mol Nutr Food Res. 2015 Jan;59(1):139–46. | Comparison with articles with similar meta-analysis results, the small sample size under the same PICO conditions. |
| Makarem N, Nicholson JM, Bandera EV, McKeown NM, Parekh N. Consumption of whole grains and cereal fiber in relation to cancer risk: a systematic review of longitudinal studies. Nutr Rev. 2016 Jun;74(6):353–73. | Comparison with articles with similar meta-analysis results, the small sample size under the same PICO conditions. |
| Ma Y, Hu M, Zhou L, Ling S, Li Y, Kong B, et al. Dietary fiber intake and risks of proximal and distal colon cancers: A meta-analysis. Medicine (Baltimore). 2018 Sep;97(36):e11678. | Comparison with articles with similar meta-analysis results, the small sample size under the same PICO conditions. |
| Yao Y, Suo T, Andersson R, Cao Y, Wang C, Lu J, et al. Dietary fibre for the prevention of recurrent colorectal adenomas and carcinomas. Cochrane Database Syst Rev. 2017 Jan 8;1(1):CD003430. | Comparison with articles with similar meta-analysis results, the small sample size under the same PICO conditions. |
| Masrul M, Nindrea RD. Dietary Fibre Protective against Colorectal Cancer Patients in Asia: A Meta-Analysis. Open Access Maced J Med Sci. 2019 May 31;7(10):1723–7. | Comparison with articles with similar meta-analysis results, the small sample size under the same PICO conditions. |
| Hoang T, Kim H, Kim J. Dietary Intake in Association with All-Cause Mortality and Colorectal Cancer Mortality among Colorectal Cancer Survivors: A Systematic Review and Meta-Analysis of Prospective Studies. Cancers (Basel). 2020 Nov 16;12(11):3391. | Comparison with articles with similar meta-analysis results, the small sample size under the same PICO conditions. |
| Asano T, McLeod RS. Dietary fibre for the prevention of colorectal adenomas and carcinomas. Cochrane Database Syst Rev. 2002;(2):CD003430. | Comparison with articles with similar meta-analysis results, the small sample size under the same PICO conditions. |
| Xu K, Sun Q, Shi Z, Zou Y, Jiang X, Wang Y, et al. A Dose-Response Meta-Analysis of Dietary Fiber Intake and Breast Cancer Risk. Asia Pac J Public Health. 2022 May;34(4):331–7. | Comparison with articles with similar meta-analysis results, the small sample size under the same PICO conditions. |
| Dong JY, He K, Wang P, Qin LQ. Dietary fiber intake and risk of breast cancer: a meta-analysis of prospective cohort studies. Am J Clin Nutr. 2011 Sep;94(3):900–5. | Comparison with articles with similar meta-analysis results, the small sample size under the same PICO conditions. |
| Mourouti N, Kontogianni MD, Papavagelis C, Panagiotakos DB. Diet and breast cancer: a systematic review. Int J Food Sci Nutr. 2015 Feb;66(1):1–42. | Comparison with articles with similar meta-analysis results, the small sample size under the same PICO conditions. |
| Coleman HG, Murray LJ, Hicks B, Bhat SK, Kubo A, Corley DA, et al. Dietary fiber and the risk of precancerous lesions and cancer of the esophagus: a systematic review and meta-analysis. Nutr Rev. 2013 Jul;71(7):474–82. | Comparison with articles with similar meta-analysis results, the small sample size under the same PICO conditions. |
| Wang CH, Qiao C, Wang RC, Zhou WP. Dietary fiber intake and pancreatic cancer risk: a meta-analysis of epidemiologic studies. Sci Rep. 2015 Jun 2;5:10834. | Comparison with articles with similar meta-analysis results, the small sample size under the same PICO conditions. |
| Nucci D, Santangelo OE, Provenzano S, Fatigoni C, Nardi M, Ferrara P, et al. Dietary Fiber Intake and Risk of Pancreatic Cancer: Systematic Review and Meta-Analysis of Observational Studies. Int J Environ Res Public Health. 2021 Nov 3;18(21):11556. | Comparison with articles with similar meta-analysis results, the small sample size under the same PICO conditions. |
| Wang RJ, Tang JE, Chen Y, Gao JG. Dietary fiber, whole grains, carbohydrate, glycemic index, and glycemic load in relation to risk of prostate cancer. Onco Targets Ther. 2015;8:2415–26. | Comparison with articles with similar meta-analysis results, the small sample size under the same PICO conditions. |
| Bandera EV, Kushi LH, Moore DF, Gifkins DM, McCullough ML. Association between dietary fiber and endometrial cancer: a dose-response meta-analysis. Am J Clin Nutr. 2007 Dec;86(6):1730–7. | Comparison with articles with similar meta-analysis results, the small sample size under the same PICO conditions. |
| Khodavandi A, Alizadeh F, Razis AFA. Association between dietary intake and risk of ovarian cancer: a systematic review and meta-analysis. Eur J Nutr. 2021 Jun;60(4):1707–36. | Comparison with articles with similar meta-analysis results, the small sample size under the same PICO conditions. |
| Huang X, Wang X, Shang J, Lin Y, Yang Y, Song Y, et al. Association between dietary fiber intake and risk of ovarian cancer: a meta-analysis of observational studies. J Int Med Res. 2018 Oct;46(10):3995–4005. | Comparison with articles with similar meta-analysis results, the small sample size under the same PICO conditions. |
| Kim Y, Je Y. Dietary fibre intake and mortality from cardiovascular disease and all cancers: A meta-analysis of prospective cohort studies. *Arch Cardiovasc Dis*. 2016;109(1):39-54. | Comparison with articles with similar meta-analysis results, the small sample size under the same PICO conditions. |
| Zheng B, Shen H, Han H, Han T, Qin Y. Dietary fiber intake and reduced risk of ovarian cancer: a meta-analysis. Nutr J. 2018 Oct 30;17(1):99. | Comparison with articles with similar meta-analysis results, the small sample size under the same PICO conditions. |
| Trock B, Lanza E, Greenwald P. Dietary fiber, vegetables, and colon cancer: critical review and meta-analyses of the epidemiologic evidence. J Natl Cancer Inst. 1990 Apr 18;82(8):650–61. | Wrong exposure or design |
| Collatuzzo G, Cortez Lainez J, Pelucchi C, et al. The association between dietary fiber intake and gastric cancer: a pooled analysis of 11 case-control studies. *Eur J Nutr*. 2024;63(5):1857-1865. | Wrong exposure or design |
| Sidahmed E, Freedland SJ, Wang M, et al. Dietary Fiber Intake and Risk of Advanced and Aggressive Forms of Prostate Cancer: A Pooled Analysis of 15 Prospective Cohort Studies. *J Acad Nutr Diet*. Published online April 16, 2024. | Wrong exposure or design |
| Arayici ME, Basbinar Y, Ellidokuz H. High and low dietary fiber consumption and cancer risk: a comprehensive umbrella review with meta-meta-analysis involving meta-analyses of observational epidemiological studies. *Crit Rev Food Sci Nutr*. Published online December 28, 2023. | Wrong exposure or design |
| Tollosa DN, James E, Hurre A, Tavener M. Cancer Survivor’s Adherence to Healthy Lifestyle Behaviours; Meeting the World Cancer Research Fund/American Institute of Cancer Research Recommendations, a Systematic Review and Meta-Analysis. World Academy of Science, Engineering and Technology, International Journal of Medical and Health Sciences. 2018 May 14; | Wrong exposure or design |
| Aldossari AA, Hann M, Sremanakova J, Sowerbutts AM, Jones D, Burden S. SUN-LB654: A Systematic Review of Dietary Intake Change in People Who Live with and Beyond Cancer. Clinical Nutrition. 2019 Sep 1;38:S302–3. | Wrong exposure or design |
| Reynolds A, Mann J, Cummings J, Winter N, Mete E, Te Morenga L. Carbohydrate quality and human health: a series of systematic reviews and meta-analyses. Lancet. 2019 Feb 2;393(10170):434–45. | Wrong exposure or design |
| Bailie L, Loughrey MB, Coleman HG. Lifestyle Risk Factors for Serrated Colorectal Polyps: A Systematic Review and Meta-analysis. Gastroenterology. 2017 Jan;152(1):92–104. | Wrong exposure or design |
| Petrick JL, Li N, McClain KM, Steck SE, Gammon MD. Dietary Risk Reduction Factors for the Barrett’s Esophagus-Esophageal Adenocarcinoma Continuum: A Review of the Recent Literature. Curr Nutr Rep. 2015 Mar 1;4(1):47–65. | Wrong exposure or design |
| Raji Lahiji M, Vafa S, de Souza RJ, Zarrati M, Sajadian A, Razmpoosh E, et al. Effect of Dietary-Based Lifestyle Modification Approaches on Anthropometric Indices and Dietary Intake Parameters in Women with Breast Cancer: A Systematic Review and Meta-Analysis of Randomized Controlled Trials. Adv Nutr. 2022 Oct 2;13(5):1974–88. | Wrong exposure or design |
| Park SH, Hoang T, Kim J. Dietary Factors and Breast Cancer Prognosis among Breast Cancer Survivors: A Systematic Review and Meta-Analysis of Cohort Studies. Cancers (Basel). 2021 Oct 23;13(21):5329. | Wrong exposure or design |
| Benisi-Kohansal S, Saneei P, Salehi-Marzijarani M, Larijani B, Esmaillzadeh A. Whole-Grain Intake and Mortality from All Causes, Cardiovascular Disease, and Cancer: A Systematic Review and Dose-Response Meta-Analysis of Prospective Cohort Studies. Adv Nutr. 2016 Nov;7(6):1052–65. | Wrong exposure or design |
| Hoang T, Kim H, Kim J. Dietary Intake in Association with All-Cause Mortality and Colorectal Cancer Mortality among Colorectal Cancer Survivors: A Systematic Review and Meta-Analysis of Prospective Studies. Cancers (Basel). 2020 Nov 16;12(11):3391. | Wrong exposure or design |
| Molina-Montes E, Ubago-Guisado E, Petrova D, Amiano P, Chirlaque MD, Agudo A, et al. The Role of Diet, Alcohol, BMI, and Physical Activity in Cancer Mortality: Summary Findings of the EPIC Study. Nutrients. 2021 Nov 28;13(12):4293. | Wrong exposure or design |
| Mourouti N, Kontogianni MD, Papavagelis C, Panagiotakos DB. Diet and breast cancer: a systematic review. Int J Food Sci Nutr. 2015 Feb;66(1):1–42. | No reported effect size |
| Schwingshackl L, Hoffmann G, Buijsse B, Mittag T, Stelmach-Mardas M, Boeing H, et al. Dietary supplements and risk of cause-specific death, cardiovascular disease, and cancer: a protocol for a systematic review and network meta-analysis of primary prevention trials. Syst Rev. 2015 Mar 26;4:34. | No reported effect size |
| Yang JJ, Yu D, Xiang YB, Blot W, Robien K, Sinha R, et al. Abstract 5253: Dietary fiber intake and lung cancer risk: A pooled analysis of 1.44 million individuals in 10 cohorts. Cancer Research. 2018 Jul 1;78(13_Supplement):5253. | Only summary information is accessible |
| Wang X, Chan A, Slattery M, Chang-Claude J, Potter J, Gallinger S, et al. Abstract PR06: Interactions between nonsteroidal anti-inflammatory drugs and other risk factors on colorectal cancer risk. Cancer Research. 2017 Feb 1;77:PR06–PR06. | Only summary information is accessible |
| Mullie P, Autier P, Boyle P. Overview of meta-analyses and pooled analyses of nutrition and breast cancer risk. Journal of Clinical Oncology. 2013 May 20;31:1519–1519. | Only summary information is accessible |
| Griffin J, Gerson L, Brockton N, et al. Can colon and rectal cancer be prevented by lifestyle modifications[C]? Diseases of the Colon and Rectum, 2020. | Only summary information is accessible |

**Supplementary Table S3.** Main results for Meta-regression model, Random effects, Z-Distribution, Log odds ratio

| Covariate | Coefficient | Z-value | 2-sided P-value |
| --- | --- | --- | --- |
| Intercept | -0.308 | -1.92 | 0.055 |
| Experimental group | 0.025 | 2.53 | 0.011 |
| Control group | -0.039 | -3.47 | 0.001 |

Proportion of total between-study variance explained by the Meta-regression mode, R^2^=0.75

**Supplementary Table S4.** Assessments of AMSTAR-2 details.

| Outcomes | Category | Assessed with | Author | Time | 1 | 2 | 3 | 4 | 5 | 6 | 7 | 8 | 9 | 10 | 11 | 12 | 13 | 14 | 15 | 16 | Level of Evidence |
| --- | --- | --- | --- | --- | --- | --- | --- | --- | --- | --- | --- | --- | --- | --- | --- | --- | --- | --- | --- | --- | --- |
| All cancer CDR | TDF | Highest versus lowest | Feifei Yao | 2023 | Yes | Yes | Yes | Partial yes | Yes | Yes | Yes | Yes | Yes | Yes | Yes | Yes | Yes | Yes | Yes | Yes | High |
| All cancer CDR | CDF | Highest versus lowest | Feifei Yao | 2023 | Yes | Yes | Yes | Partial yes | Yes | Yes | Yes | Yes | Yes | Yes | Yes | Yes | Yes | Yes | Yes | Yes | High |
| All cancer CDR | IDF | Highest versus lowest | Feifei Yao | 2023 | Yes | Yes | Yes | Partial yes | Yes | Yes | Yes | Yes | Yes | Yes | Yes | Yes | Yes | Yes | Yes | Yes | High |
| All cancer CDR | SDF | Highest versus lowest | Feifei Yao | 2023 | Yes | Yes | Yes | Partial yes | Yes | Yes | Yes | Yes | Yes | Yes | Yes | Yes | Yes | Yes | Yes | Yes | High |
| All cancer ACM | CDF | Highest versus lowest | Hajishafiee | 2016 | Yes | No | Yes | Partial yes | Yes | Yes | No | Partial yes | No | Yes | Yes | No | No | Yes | Yes | Yes | Very low |
| Esophageal adenocarcinoma IR | TDF | Highest versus lowest | Lingli Sun | 2017 | Yes | Partial yes | Yes | Partial yes | Yes | Yes | No | Partial yes | Partial yes | No | Yes | Yes | Yes | Yes | Yes | Yes | Very low |
| Esophageal squamous cell carcinoma IR | TDF | Highest versus lowest | Lingli Sun | 2017 | Yes | Partial yes | Yes | Partial yes | Yes | Yes | No | Partial yes | Partial yes | No | Yes | Yes | Yes | Yes | Yes | Yes | Very low |
| Barrett’s esophagus and esophageal cancer IR | TDF | Highest versus lowest | Lingli Sun | 2017 | Yes | Partial yes | Yes | Partial yes | Yes | Yes | No | Partial yes | Partial yes | No | Yes | Yes | Yes | Yes | Yes | Yes | Very low |
| Gastric cancer IR | TDF | Highest versus lowest | Zhizhong Zhang | 2013 | Yes | No | No | Partial yes | Yes | Yes | No | Partial yes | Yes | Yes | Yes | No | Yes | No | Yes | No | Very low |
| Colon cancer IR | TDF | Highest versus lowest | Vincenza Gianfredi | 2018 | Yes | No | No | Partial yes | Yes | Yes | No | Partial yes | Yes | No | Yes | No | Yes | No | Yes | No | Very low |
| Colorectal cancer IR | TDF | Highest versus lowest | Dagfinn Aune | 2011 | Yes | Partial yes | Yes | Partial yes | Yes | Yes | No | Yes | No | Yes | Yes | No | No | No | Yes | Yes | Very low |
| Colorectal cancer ACM | TDF | Highest versus lowest | Jing Zhao | 2022 | Yes | Yes | Yes | Yes | Yes | Yes | Yes | Yes | Yes | Yes | Yes | Yes | Yes | Yes | Yes | Yes | High |
| Colorectal cancer CDR | TDF | Highest versus lowest | Jing Zhao | 2022 | Yes | Yes | Yes | Yes | Yes | Yes | Yes | Yes | Yes | Yes | Yes | Yes | Yes | Yes | Yes | Yes | High |
| Colorectal cancer CDR | SDF | Highest versus lowest | Fatemeh Ramezani | 2023 | Yes | Yes | Yes | Yes | Yes | Yes | Yes | Yes | Yes | Yes | Yes | Yes | Yes | Yes | Yes | Yes | High |
| Colorectal cancer CDR | IDF | Highest versus lowest | Fatemeh Ramezani | 2023 | Yes | Yes | Yes | Yes | Yes | Yes | Yes | Yes | Yes | Yes | Yes | Yes | Yes | Yes | Yes | Yes | High |
| Colorectal cancer CDR | VDF | Highest versus lowest | Fatemeh Ramezani | 2023 | Yes | Yes | Yes | Yes | Yes | Yes | Yes | Yes | Yes | Yes | Yes | Yes | Yes | Yes | Yes | Yes | High |
| Colorectal cancer CDR | FDF | Highest versus lowest | Fatemeh Ramezani | 2023 | Yes | Yes | Yes | Yes | Yes | Yes | Yes | Yes | Yes | Yes | Yes | Yes | Yes | Yes | Yes | Yes | High |
| Colorectal cancer CDR | LDF | Highest versus lowest | Fatemeh Ramezani | 2023 | Yes | Yes | Yes | Yes | Yes | Yes | Yes | Yes | Yes | Yes | Yes | Yes | Yes | Yes | Yes | Yes | High |
| Colorectal adenoma IR | TDF | Highest versus lowest | Daniele Nucci | 2021 | Yes | No | No | Partial yes | Yes | Yes | Yes | Yes | Partial yes | Yes | Yes | No | No | Yes | Yes | Yes | Low |
| Colorectal cancer IR | CDF | Highest versus lowest | Dagfinn Aune | 2011 | Yes | Partial yes | Yes | Partial yes | Yes | Yes | No | Yes | No | Yes | Yes | No | No | No | Yes | Yes | Very low |
| Colorectal adenoma IR | CDF | Highest versus lowest | Qiwen Ben | 2014 | Yes | No | Yes | Partial yes | Yes | Yes | No | Yes | Yes | Yes | Yes | Yes | Yes | Yes | Yes | Yes | Very low |
| Colorectal cancer IR | VDF | Highest versus lowest | Dagfinn Aune | 2011 | Yes | Partial yes | Yes | Partial yes | Yes | Yes | No | Yes | No | Yes | Yes | No | No | No | Yes | Yes | Very low |
| Colorectal adenoma IR | VDF | Highest versus lowest | Qiwen Ben | 2014 | Yes | No | Yes | Partial yes | Yes | Yes | No | Yes | Yes | Yes | Yes | Yes | Yes | Yes | Yes | Yes | Very low |
| Colorectal cancer IR | LDF | Highest versus lowest | Dagfinn Aune | 2011 | Yes | Partial yes | Yes | Partial yes | Yes | Yes | No | Yes | No | Yes | Yes | No | No | No | Yes | Yes | Very low |
| Colorectal adenoma IR | FDF | Highest versus lowest | Qiwen Ben | 2014 | Yes | No | Yes | Partial yes | Yes | Yes | No | Yes | Yes | Yes | Yes | Yes | Yes | Yes | Yes | Yes | Very low |
| Colorectal cancer IR | FDF | Highest versus lowest | Dagfinn Aune | 2011 | Yes | Partial yes | Yes | Partial yes | Yes | Yes | No | Yes | No | Yes | Yes | No | No | No | Yes | Yes | Very low |
| Colorectal cancer IR | SDF | Highest versus lowest | Mehmet Emin Arayici | 2022 | Yes | No | Yes | Partial yes | Yes | Yes | No | Partial yes | Yes | Yes | Yes | No | Yes | No | Yes | Yes | Very low |
| Colorectal cancer IR | IDF | Highest versus lowest | Mehmet Emin Arayici | 2022 | Yes | No | Yes | Partial yes | Yes | Yes | No | Partial yes | Yes | Yes | Yes | No | Yes | No | Yes | Yes | Very low |
| liver cancer IR | TDF | Highest versus lowest | Cody Z. Watling | 2024 | Yes | Yes | Yes | No | No | No | No | Yes | Yes | No | Yes | Yes | Yes | Yes | Yes | No | Very low |
| liver cancer IR(ICC) | TDF | Highest versus lowest | Cody Z. Watling | 2024 | Yes | Yes | Yes | No | No | No | No | Yes | Yes | No | Yes | Yes | Yes | Yes | Yes | No | Very low |
| liver cancer IR(HCC) | TDF | Highest versus lowest | Cody Z. Watling | 2024 | Yes | Yes | Yes | No | No | No | No | Yes | Yes | No | Yes | Yes | Yes | Yes | Yes | No | Very low |
| Rectal cancer IR | TDF | Highest versus lowest | Vincenza Gianfredi | 2019 | Yes | No | No | Partial yes | Yes | Yes | No | Partial yes | Yes | Yes | Yes | Yes | No | No | Yes | Yes | Very low |
| Breast cancer IR | TDF | Highest versus lowest | Sumei Chen | 2016 | Yes | No | No | Partial yes | Yes | Yes | No | Partial yes | No | No | No | No | No | Yes | Yes | Yes | Very low |
| Breast cancer ACM | TDF | Highest versus lowest | Ahmad Jayedi | 2021 | Yes | No | No | Partial yes | Yes | Yes | Yes | Yes | No | Yes | Yes | No | No | No | No | Yes | Very low |
| Breast cancer CDR | TDF | Highest versus lowest | Ahmad Jayedi | 2021 | Yes | No | No | Partial yes | Yes | Yes | Yes | Yes | No | Yes | Yes | No | No | No | No | Yes | Very low |
| Breast cancer IR | CDF | Highest versus lowest | Maryam S. Farvid | 2020 | Yes | Partial yes | Yes | No | No | No | No | Yes | No | Yes | Yes | Yes | Yes | Yes | Yes | Yes | Very low |
| Breast cancer IR | VDF | Highest versus lowest | Maryam S. Farvid | 2020 | Yes | Partial yes | Yes | No | No | No | No | Yes | No | Yes | Yes | Yes | Yes | Yes | Yes | Yes | Very low |
| Breast cancer IR | LDF | Highest versus lowest | Maryam S. Farvid | 2020 | Yes | Partial yes | Yes | No | No | No | No | Yes | No | Yes | Yes | Yes | Yes | Yes | Yes | Yes | Very low |
| Breast cancer IR | FDF | Highest versus lowest | Maryam S. Farvid | 2020 | Yes | Partial yes | Yes | No | No | No | No | Yes | No | Yes | Yes | Yes | Yes | Yes | Yes | Yes | Very low |
| Breast cancer IR | SDF | Highest versus lowest | Maryam S. Farvid | 2020 | Yes | Partial yes | Yes | No | No | No | No | Yes | No | Yes | Yes | Yes | Yes | Yes | Yes | Yes | Very low |
| Breast cancer IR | IDF | Highest versus lowest | Maryam S. Farvid | 2020 | Yes | Partial yes | Yes | No | No | No | No | Yes | No | Yes | Yes | Yes | Yes | Yes | Yes | Yes | Very low |
| Ovarian cancer IR | TDF | Highest versus lowest | Hui Xu | 2018 | Yes | No | No | Yes | Yes | Yes | No | Yes | No | Yes | Yes | Yes | No | Yes | No | Yes | Very low |
| Endometrial Cancer IR | CDF | Highest versus lowest | Kangning Chen | 2018 | Yes | No | No | Partial yes | Yes | Yes | No | Partial yes | Partial yes | Yes | Yes | Yes | No | No | Yes | Yes | Very low |
| Endometrial Cancer IR | VDF | Highest versus lowest | Kangning Chen | 2018 | Yes | No | No | Partial yes | Yes | Yes | No | Partial yes | Partial yes | Yes | Yes | Yes | No | No | Yes | Yes | Very low |
| Endometrial cancer IR | TDF | Highest versus lowest | Hengjie Li | 2020 | Yes | No | No | Partial yes | Yes | Yes | No | Partial yes | Partial yes | Yes | Yes | Yes | Yes | No | Yes | Yes | Critically low |
| Prostate cancer IR | TDF | Highest versus lowest | Tao Sheng | 2015 | No | No | No | Partial yes | Yes | Yes | Yes | Yes | No | No | Yes | No | No | Yes | No | No | Very low |
| Renal cell carcinoma IR | TDF | Highest versus lowest | Xin Xu | 2019 | Yes | No | No | Partial yes | Yes | Yes | Partial yes | No | Yes | Yes | Yes | Yes | Yes | Yes | Yes | Yes | Low |
| Renal cell carcinoma IR | CDF | Highest versus lowest | Tian-bao Huang | 2014 | Yes | No | Yes | Partial yes | Yes | Yes | No | Partial yes | Partial yes | No | Yes | Yes | No | No | No | Yes | Very low |
| Renal cell carcinoma IR | VDF | Highest versus lowest | Tian-bao Huang | 2014 | Yes | No | Yes | Partial yes | Yes | Yes | No | Partial yes | Partial yes | No | Yes | Yes | No | No | No | Yes | Very low |
| Renal cell carcinoma IR | LDF | Highest versus lowest | Tian-bao Huang | 2014 | Yes | No | Yes | Partial yes | Yes | Yes | No | Partial yes | Partial yes | No | Yes | Yes | No | No | No | Yes | Very low |
| Renal cell carcinoma IR | FDF | Highest versus lowest | Tian-bao Huang | 2014 | Yes | No | Yes | Partial yes | Yes | Yes | No | Partial yes | Partial yes | No | Yes | Yes | No | No | No | Yes | Very low |
| Bladder cancer IR | TDF | Highest versus lowest | Evan YW Yu | 2020 | Yes | No | Yes | Partial yes | Yes | Yes | No | Partial yes | Yes | Yes | Yes | No | No | No | No | Yes | Very low |
| Esophageal eancer IR | TDF | 10 g/day increment | Lingli Sun | 2017 | Yes | Partial yes | Yes | Partial yes | Yes | Yes | No | Partial yes | Partial yes | No | Yes | Yes | Yes | Yes | Yes | Yes | Very low |
| Gastric cancer IR | TDF | 10 g/day increment | Zhizhong Zhang | 2013 | Yes | No | No | Partial yes | Yes | Yes | No | Partial yes | Yes | Yes | Yes | No | Yes | No | Yes | No | Very low |
| Colorectal cancer IR | TDF | 10 g/day increment | Dagfinn Aune | 2011 | Yes | Partial yes | Yes | Partial yes | Yes | Yes | No | Yes | No | Yes | Yes | No | No | No | Yes | Yes | Very low |
| Colorectal cancer IR | CDF | 10 g/day increment | Dagfinn Aune | 2011 | Yes | Partial yes | Yes | Partial yes | Yes | Yes | No | Yes | No | Yes | Yes | No | No | No | Yes | Yes | Very low |
| Colorectal cancer IR | VDF | 10 g/day increment | Dagfinn Aune | 2011 | Yes | Partial yes | Yes | Partial yes | Yes | Yes | No | Yes | No | Yes | Yes | No | No | No | Yes | Yes | Very low |
| Colorectal cancer IR | LDF | 10 g/day increment | Dagfinn Aune | 2011 | Yes | Partial yes | Yes | Partial yes | Yes | Yes | No | Yes | No | Yes | Yes | No | No | No | Yes | Yes | Very low |
| Colorectal adenomas RR | CDF | 10 g/day increment | Hannah Oh | 2019 | Yes | No | No | Yes | Yes | No | No | Yes | Partial yes | Yes | Yes | Yes | Yes | Yes | Yes | Yes | Low |
| Colorectal adenomas RR | VDF | 10 g/day increment | Hannah Oh | 2019 | Yes | No | No | Yes | Yes | No | No | Yes | Partial yes | Yes | Yes | Yes | Yes | Yes | Yes | Yes | Low |
| Colorectal adenomas RR | FDF | 10 g/day increment | Hannah Oh | 2019 | Yes | No | No | Yes | Yes | No | No | Yes | Partial yes | Yes | Yes | Yes | Yes | Yes | Yes | Yes | Low |
| Breast cancer IR | TDF | 10 g/day increment | Sumei Chen | 2016 | Yes | No | No | Partial yes | Yes | Yes | No | Partial yes | No | No | No | No | No | Yes | Yes | Yes | Very low |
| Breast cancer IR | CDF | 10 g/day increment | Dagfinn Aune | 2012 | Yes | No | Yes | Partial yes | No | No | Yes | No | Partial yes | Yes | Yes | Yes | Yes | Yes | Yes | Yes | Very low |
| Ovarian cancer IR | TDF | 5 g/day increment | Hui Xu | 2018 | Yes | No | No | Yes | Yes | Yes | No | Yes | No | Yes | Yes | Yes | No | Yes | No | Yes | Very low |
| Renal cell carcinoma IR | LDF | 2.5 g/day increment | Tian-bao Huang | 2014 | Yes | No | Yes | Partial yes | Yes | Yes | No | Partial yes | Partial yes | No | Yes | Yes | No | No | No | Yes | Very low |
| Renal cell carcinoma IR | FDF | 2.5 g/day increment | Tian-bao Huang | 2014 | Yes | No | Yes | Partial yes | Yes | Yes | No | Partial yes | Partial yes | No | Yes | Yes | No | No | No | Yes | Very low |
| Pancreatic cancer IR | TDF | 10 g/day increment | Qiqi Mao | 2017 | Yes | Yes | No | Yes | Yes | Yes | No | Yes | Yes | No | Yes | Yes | Yes | Yes | Yes | Yes | Medium |
| Bladder cancer IR | TDF | 5 g/day increment | Evan YW Yu | 2020 | Yes | No | Yes | Partial yes | Yes | Yes | No | Partial yes | Yes | Yes | Yes | No | No | No | No | Yes | Very low |
| All cancer CDR | TDF | 10 g/day increment | Feifei Yao | 2023 | Yes | Yes | Yes | Partial yes | Yes | Yes | Yes | Yes | Yes | Yes | Yes | Yes | Yes | Yes | Yes | Yes | High |
| Liver cancer IR | TDF | 10 g/day increment | Cody Z. Watling | 2024 | Yes | Yes | Yes | No | No | No | No | Yes | Yes | No | Yes | Yes | Yes | Yes | Yes | No | Very low |

RR= relapse rate; IR= incident rate; CDR = crude death rate; ACM = all-cause mortality rate; TDF=total dietary fiber; CDF=cereal dietary fiber; VDF=vegetable dietary fiber; LDF=legume dietary fiber; FDF=fruit dietary fiber; SDF=soluble dietary fiber; IDF=intolerable dietary fiber;

1.Did the research questions and inclusion criteria for the review include the components of PICO? 2. Did the report of the review contain an explicit statement that the review methods were established prior to the conduct of the review and did the report justify any significant deviations from the protocol 3. Did the review authors explain their selection of the study designs for inclusion in the review? 4. Did the review authors use a comprehensive literature search strategy? 5. Did the review authors perform study selection in duplicate? 6. Did the review authors perform data extraction in duplicate? 7. Did the review authors provide a list of excluded studies and justify the exclusions? 8. Did the review authors describe the included studies in adequate detail? 9. Did the review authors use a satisfactory technique for assessing the risk of bias (RoB) in individual studies that were included in the review? 10. Did the review authors report on the sources of funding for the studies included in the review?11. If meta-analysis was performed did the review authors use appropriate methods for statistical combination of results? 12.If meta-analysis was performed, did the review authors assess the potential impact of RoB in individual studies on the results of the meta-analysis or other evidence synthesis? 13. Did the review authors account for RoB in individual studies when interpreting/ discussing the results of the review? 14. Did the review authors provide a satisfactory explanation for, and discussion of, any heterogeneity observed in the results of the review? 15. If they performed quantitative synthesis did the review authors carry out an adequate investigation of publication bias (small study bias) and discuss its likely impact on the results of the review? 16. Did the review authors report any potential sources of conflict of interest, including any funding they received for conducting the review?

**
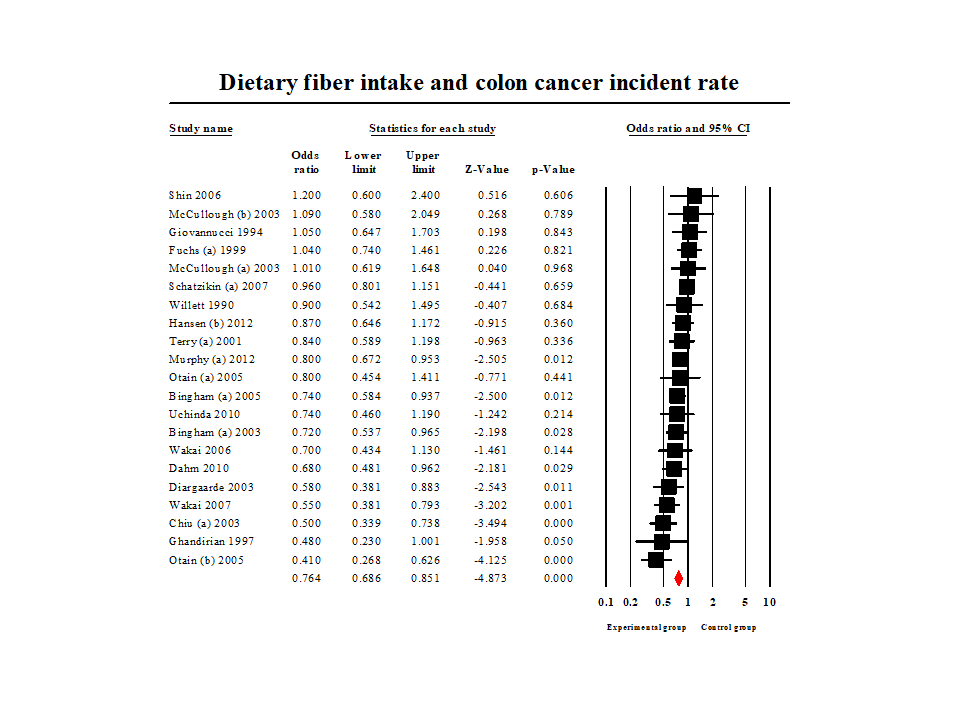
**

**Figure S1.** **Relationship between Dietary Fiber Intake and Colon Cancer Incidence Rate**
